# Supplementary material for: Giant optical polarisation rotations induced by a single quantum dot spin
Source: Nat Commun. 2024 Jan 18;15:598. doi: 10.1038/s41467-023-44651-8 (PMC10796934; doi:10.1038/s41467-023-44651-8)
Supplement: Supplementary file 1 — Supplementary Information [file 41467_2023_44651_MOESM1_ESM.pdf]

Supplementary Notes: Giant optical polarisation rotations induced by a single quantum dot spin

E. Mehdi,[1,2] M. Gundin,[1] C. Millet,[1] N. Somaschi,[3] A. Lemaître,[1] I. Sagnes,[1] L. Le Gratiet,[1] D. A. Fioretto,[1,3] N. Belabas,[1] O. Krebs,[1] P. Senellart,[1] and L. Lanco[1,2,4,5]

[1] *Université Paris-Saclay, CNRS, Centre de Nanosciences et de Nanotechnologies, 91120, Palaiseau, France*
[2] *Université Paris Cité, Centre de Nanosciences et de Nanotechnologies, 91120, Palaiseau, France*
[3] *Quandela, 7 rue Leonard de Vinci, 91300, Massy, France*
[4] *Institut Universitaire de France (IUF), 75005, Paris, France*
[5] *Corresponding author: loic.lanco@c2n.upsaclay.fr*

Contents

Supplementary Note 1 - Master equation formalism 1

Supplementary Note 2 - Analytical and numerical predictions for the reflected polarisation states and intensities 4

Supplementary Note 3 - Parameter estimation using only intensity measurements 7

Supplementary Note 4 - Exact numerical simulations including the full system dynamics 8

Supplementary Note 5 - Co-tunneling regime and bias dependence 10

Supplementary Note 6 - Analysis of spin-photon mapping in the limit of large birefringence 12

Supplementary Note 7 - Extended data 14
- Cavity birefringence 14
- Residual cavity-induced polarisation rotations 15
- Measurements of average and extrapolated Stokes parameters 16
- Simulations of the possible output polarisation states 17

References 18

Supplementary Note 1 - Master equation formalism

In this work, all analytical and numerical calculations are based on the resolution of the master equation, which describes the evolution of the system's density matrix $\hat{\rho}$, in the following form:

$$\frac{d\hat{\rho}(t)}{dt} = \frac{i}{\hbar}\left[\hat{\rho}(t), \hat{H}\right] + \sum_k \hat{L}_k[\hat{\rho}(t)] \tag{1}$$

In this master equation, all terms are defined as functions of some elementary operators. This includes $\hat{a}_X$ and $\hat{a}_X^\dagger$, the annihilation and creation operators for X-polarised photons in the cavity modes $X = H, V$. We also use $\hat{\sigma}_{R/L}$, the de-excitation operators describing the trion decay after the emission of a R/L-polarised photon, defined as $\hat{\sigma}_R = |\uparrow\rangle\langle\uparrow\downarrow\Uparrow|$ and $\hat{\sigma}_L = |\downarrow\rangle\langle\downarrow\uparrow\Downarrow|$. We also define $\hat{\sigma}_{H/V}$ as

$$\hat{\sigma}_H = \frac{\hat{\sigma}_R + \hat{\sigma}_L}{\sqrt{2}} \text{ and } \hat{\sigma}_V = i\frac{\hat{\sigma}_R - \hat{\sigma}_L}{\sqrt{2}}.$$

In addition, we introduce $\hat{S}_z^{(\mathrm{e})}$ and $\hat{S}_z^{(\mathrm{h})}$ the spin operators in the growth direction, for the electron spin (e) and for the hole (h) pseudospin in the trion state ($\hbar = 1$ units are used in all this work):

$$\hat{S}_z^{(\mathrm{e})} = \frac{1}{2}\left(|\uparrow\rangle\langle\uparrow| - |\downarrow\rangle\langle\downarrow|\right)$$
$$\hat{S}_z^{(\mathrm{h})} = \frac{1}{2}\left(|\uparrow\downarrow\Uparrow\rangle\langle\uparrow\downarrow\Uparrow| - |\downarrow\uparrow\Downarrow\rangle\langle\downarrow\uparrow\Downarrow|\right)$$

Furthermore, using the input-output formalism [1], we define the input (resp. output) external field operator $\hat{b}_{\mathrm{in},X}$ (resp. $\hat{b}_{\mathrm{out,X}}$), which correspond to the incident (resp. escaping) external fields that couples through the top mirror with the $X = H/V$ cavity mode. Since a coherent input laser is used, the operator $\hat{b}_{\mathrm{in},X}$ can be replaced by

$$\hat{b}_{\mathrm{in},X} = b_{\mathrm{in},X}\hat{I}, \tag{2}$$

with $\hat{I}$ the identity operator [2]. The output field operators $\hat{b}_{\mathrm{out,X}}$ can then be deduced using the input-output relation [1]:

$$\hat{b}_{\mathrm{out,X}} = \hat{b}_{\mathrm{in},X} + \sqrt{\kappa_{\mathrm{top},X}}\,\hat{a}_X, \tag{3}$$

with $\kappa_{\mathrm{top},X}$ the cavity damping rate from the top mirror for mode $X = H/V$. From these operators one can also define:

$$\begin{aligned} \hat{b}_{\mathrm{out},D} &= \frac{\hat{b}_{\mathrm{out},H} + \hat{b}_{\mathrm{out},V}}{\sqrt{2}} \qquad & \hat{b}_{\mathrm{out},R} &= \frac{\hat{b}_{\mathrm{out},H} + i\,\hat{b}_{\mathrm{out},V}}{\sqrt{2}} \\ \hat{b}_{\mathrm{out},A} &= \frac{\hat{b}_{\mathrm{out},H} - \hat{b}_{\mathrm{out},V}}{\sqrt{2}} \qquad & \hat{b}_{\mathrm{out},L} &= \frac{\hat{b}_{\mathrm{out},H} - i\,\hat{b}_{\mathrm{out},V}}{\sqrt{2}} \end{aligned} \tag{4}$$

All the useful operators being defined, one can also express the total Hamiltonian $\hat{H}$ governing the coherent processes:

$$\hat{H} = \sum_{X=H,V}\left(\hat{H}_{\mathrm{QD},X} + \hat{H}_{\mathrm{cav},X} + \hat{H}_{\mathrm{int},X} + \hat{H}_{\mathrm{pump},X}\right) + \hat{H}_B \tag{5}$$

In this Hamiltonian:

- $\hat{H}_{\mathrm{QD},X}$ is the QD Hamiltonian written in the frame rotating at frequency ω_{laser}, with :

  $$\hat{H}_{\mathrm{QD},X} = (\omega_{\mathrm{QD}} - \omega_{\mathrm{laser}})\hat{\sigma}_X^\dagger\hat{\sigma}_X,$$

  where ω_{QD} is the quantum dot transition energy in absence of applied magnetic field.

- $\hat{H}_{\mathrm{cav},X}$ is the cavity Hamiltonian, in the same rotating frame:

  $$\hat{H}_{\mathrm{cav},X} = (\omega_{\mathrm{cav},X} - \omega_{\mathrm{laser}})\hat{a}_X^\dagger\hat{a}_X,$$

  where $\omega_{\mathrm{cav},H}$ and $\omega_{\mathrm{cav},V}$ are discussed in Supplementary Note 7 - cavity birefringence.

- $\hat{H}_{\mathrm{int},X}$ is the QD-cavity interaction Hamiltonian:

  $$\hat{H}_{\mathrm{int},X} = ig(\hat{\sigma}_X^\dagger\hat{a}_X - \hat{\sigma}_X\hat{a}_X^\dagger),$$

  with g the light-matter coupling.

- $\hat{H}_{\text{pump},X}$ is the pumping Hamiltonian:

$$\hat{H}_{\text{pump},X} = -i\sqrt{\kappa_{\text{top},X}}(\hat{b}_{\text{in},X}\hat{a}_X^\dagger - \hat{b}_{\text{in},X}^\dagger \hat{a}_X)$$

- $\hat{H}_B$ describes the response to an external Faraday magnetic field:

$$\hat{H}_B = B_z\mu_B\left(g_{h,\text{long}}\hat{S}_z^{(\text{h})} + g_{e,\text{long}}\hat{S}_z^{(\text{h})}\right) + \delta_{\text{diamagnetic}}B_z^2\left(\sigma_L^\dagger\sigma_L + \sigma_R^\dagger\sigma_R\right)$$

  with B_z the value of the applied magnetic field (denoted B in the main text), μ_B the Bohr magneton, $g_{h,\text{long}}$ and $g_{e,\text{long}}$ the hole pseudospin and electron spin longitudinal Landé factors, and $\delta_{\text{diamagnetic}}$ the diamagnetic shift constant. As discussed in the main text, the effect of this magnetic field is simply to shift the transition energies $\omega_{\text{QD}}^\uparrow$ and $\omega_{\text{QD}}^\downarrow$, and lift their degeneracy.

Furthermore, the incoherent processes are described by the generic superoperator defined as

$$\hat{L}_k[\hat{\rho}] = \hat{C}_k\hat{\rho}\hat{C}_k^\dagger - \frac{1}{2}\left(\hat{C}_k^\dagger\hat{C}_k\hat{\rho} + \hat{\rho}\hat{C}_k^\dagger\hat{C}_k\right) \tag{6}$$

with $\hat{C}_k$ the collapse operator representing a given incoherent process:

- The cavity decay processes leading X-polarised intracavity photons to escape the cavity (for $X = H$ or V) are described by collapse operators $\hat{C}_{\text{cav},X}$:

$$\hat{C}_{\text{cav},X} = \sqrt{\kappa_X}\hat{a}_X$$

  with κ_X the total cavity intensity decay rate for mode X.

- The QD decay processes leading to spontaneous emission of photons in any leaky mode other than the cavity mode are described by collapse operators $\hat{C}_{\text{sp},X}$:

$$\hat{C}_{\text{sp},X} = \sqrt{\gamma_{\text{sp}}}\hat{\sigma}_X$$

  with γ_{sp} the spontaneous emission rate in leaky modes.

The different parameters introduced here also allow defining dimensionless quantities, some of them being mentioned in the main text. This includes:

- The cooperativities that the device would display, for each mode H, V, in absence of environmental noise:

$$C_H = \frac{2g^2}{\kappa_H\,\gamma_{sp}} \qquad C_V = \frac{2g^2}{\kappa_V\,\gamma_{sp}}$$

  (here $C_{H,V} \approx 8 \pm 2$ for both modes, according to the values discussed in the main text)

- The top-mirror output coupling efficiency, which describes the probability that an internal photon in the H, V cavity mode escapes it through the top-mirror:

$$\eta_{\text{top},H} = \frac{\kappa_{\text{top},H}}{\kappa_H} \qquad \eta_{\text{top},V} = \frac{\kappa_{\text{top},V}}{\kappa_V}$$

  (here $\eta_{\text{top},H} = \eta_{\text{top},V} = 0.635 \pm 0.01$ for both modes, see Supplementary Note 7.1)

- The normalized detunings between the laser and the H, V cavity mode:

$$\Delta_H = 2\frac{\omega_{\text{laser}} - \omega_{\text{cav},H}}{\kappa_H} \qquad \Delta_V = 2\frac{\omega_{\text{laser}} - \omega_{\text{cav},V}}{\kappa_V}$$

- The normalized detunings between the laser and each of the two quantum dot transitions, $|\uparrow\rangle - |\uparrow\downarrow\Uparrow\rangle$ and $|\downarrow\rangle - |\downarrow\uparrow\Downarrow\rangle$:

$$\Delta_{QD}^\uparrow = 2\frac{\omega_{\text{laser}} - \omega_{QD}^\uparrow}{\gamma_{sp}} \qquad \Delta_{QD}^\downarrow = 2\frac{\omega_{\text{laser}} - \omega_{QD}^\downarrow}{\gamma_{sp}}$$

Supplementary Note 2 - Analytical and numerical predictions for the reflected polarisation states and intensities

In this section, we first discuss the reflection coefficients and polarisation states description in the semi-classical regime. Such regime corresponds to the case where the output field can be considered as coherent, fully characterized by its average value $b_{\text{out},X} = \langle \hat{b}_{\text{out},X} \rangle$, just as the input field is fully characterized by $b_{\text{in},X} = \langle \hat{b}_{\text{in},X} \rangle$. This is possible when the QD resonance fluorescence is coherent, emitting at the exact frequency as the incoming laser [3], the incoherent contribution being neglected. Such an approximation becomes valid, in particular, in the linear low-power regime, and as long as no fluctuations occur (the effect of slow fluctuations, including spectral fluctuations and spin-flip or charge/escape mechanisms, is described later on).

To derive analytical formulae, one can solve the optical Bloch equations in the stationary regime for the QD and cavity operators, namely $\langle \dot{\hat{a}}_{H/V} \rangle = 0$ and $\langle \dot{\hat{\sigma}}_{R/L} \rangle = 0$. Note that we choose different polarisation bases for the quantum dot operators and the cavity ones. The circular right and left polarisations (R and L) are indeed the proper basis to describe the QD, as they are given by the selection rules in the studied configuration. On the other hand, the H and V eigenmodes correspond to the proper basis to describe the cavity.

The optical Bloch equations are found by multiplying all terms of the master equation by $\hat{a}_{H/V}$ ($\hat{\sigma}_{R/L}$ respectively) on the left, and by taking the trace. One obtains:

$$\langle \dot{\hat{a}}_H \rangle = -i(\omega_{\text{cav},H} - \omega_{\text{laser}}) \langle \hat{a}_H \rangle - \sqrt{\kappa_{\text{top},H}} \langle \hat{b}_{\text{in},H} \rangle - \frac{\kappa_H}{2} \langle \hat{a}_H \rangle - \frac{g}{\sqrt{2}} (\langle \hat{\sigma}_R \rangle + \langle \hat{\sigma}_L \rangle)$$

$$\langle \dot{\hat{a}}_V \rangle = -i(\omega_{\text{cav},V} - \omega_{\text{laser}}) \langle \hat{a}_V \rangle - \sqrt{\kappa_{\text{top},V}} \langle \hat{b}_{\text{in},V} \rangle - \frac{\kappa_V}{2} \langle \hat{a}_V \rangle - i\frac{g}{\sqrt{2}} (\langle \hat{\sigma}_L \rangle - \langle \hat{\sigma}_R \rangle)$$

together with:

$$\begin{aligned} \langle \dot{\hat{\sigma}}_{R/L} \rangle = &- i(\omega_{\text{QD}}^{\uparrow/\downarrow} - \omega_{\text{laser}}) \langle \hat{\sigma}_{R/L} \rangle - \frac{\gamma_{sp}}{2} \langle \hat{\sigma}_{R/L} \rangle \\ &- \frac{g}{\sqrt{2}} \left(\langle \hat{\sigma}^\dagger_{R/L} \hat{\sigma}_{R/L} \hat{a}_H \rangle - \langle \hat{\sigma}_{R/L} \hat{\sigma}^\dagger_{R/L} \hat{a}_H \rangle \pm i \langle \hat{\sigma}^\dagger_{R/L} \hat{\sigma}_{R/L} \hat{a}_V \rangle \mp i \langle \hat{\sigma}_{R/L} \hat{\sigma}^\dagger_{R/L} \hat{a}_V \rangle \right) \end{aligned}$$

The semiclassical approximation, in such a case, consists in replacing the average value of product operators, such as $\langle \hat{A}\hat{B} \rangle$, by the product of average values $\langle \hat{A} \rangle \langle \hat{B} \rangle$ [2]. For a spin perfectly initialised in the state $|\uparrow\rangle$, and in the low-power limit, $P(|\uparrow\rangle) = \langle \hat{\sigma}_R \hat{\sigma}^\dagger_R \rangle_\uparrow = 1$, all other state populations being zero. Simplified equations are then obtained:

$$\langle \dot{\hat{\sigma}}_R \rangle_\uparrow = -i(\omega_{\text{QD}}^{\uparrow} - \omega_{\text{laser}}) \langle \hat{\sigma}_R \rangle_\uparrow - \frac{\gamma_{\text{sp}}}{2} \langle \hat{\sigma}_R \rangle_\uparrow + \frac{g}{\sqrt{2}} \left(\langle \hat{a}_H \rangle_\uparrow + i \langle \hat{a}_V \rangle_\uparrow \right)$$

$$\langle \dot{\hat{\sigma}}_L \rangle_\uparrow = -i(\omega_{\text{QD}}^{\downarrow} - \omega_{\text{laser}}) \langle \hat{\sigma}_L \rangle_\uparrow - \frac{\gamma_{\text{sp}}}{2} \langle \hat{\sigma}_L \rangle_\uparrow$$

Conversely, if the spin is initialized in state $|\downarrow\rangle$:

$$\langle \dot{\hat{\sigma}}_R \rangle_\downarrow = -i(\omega_{\text{QD}}^{\uparrow} - \omega_{\text{laser}}) \langle \hat{\sigma}_R \rangle_\downarrow - \frac{\gamma_{\text{sp}}}{2} \langle \hat{\sigma}_R \rangle_\downarrow$$

$$\langle \dot{\hat{\sigma}}_L \rangle_\downarrow = -i(\omega_{\text{QD}}^{\downarrow} - \omega_{\text{laser}}) \langle \hat{\sigma}_L \rangle_\downarrow - \frac{\gamma_{\text{sp}}}{2} \langle \hat{\sigma}_L \rangle_\downarrow + \frac{g}{\sqrt{2}} \left(\langle \hat{a}_H \rangle_\uparrow - i \langle \hat{a}_V \rangle_\uparrow \right)$$

Solving the above equations in the stationary regime, one can deduce the average values $\langle \hat{a}_{H/V} \rangle_{\uparrow/\downarrow}$. Finally, one can derive the relations between the input and output fields [4], using their definition given by (2) and (3). One obtains:

$$b^{\uparrow/\downarrow}_{\text{out},H} = r^{\uparrow/\downarrow}_{H\to H}\, b_{\text{in},H} + r^{\uparrow/\downarrow}_{V\to H}\, b_{\text{in},V}$$
$$b^{\uparrow/\downarrow}_{\text{out},V} = r^{\uparrow/\downarrow}_{H\to V}\, b_{\text{in},H} + r^{\uparrow/\downarrow}_{V\to V}\, b_{\text{in},V} \tag{7}$$

with:

$$r^{\uparrow/\downarrow}_{V\to H} = \pm 2\, i\, \sqrt{\eta_{\text{top,H}}\, \eta_{\text{top,V}}}\, \frac{\sqrt{C_H\, C_V}}{\left(\left(1 - i\Delta^{\uparrow/\downarrow}_{QD}\right) + \frac{C_H}{1-i\Delta_H} + \frac{C_V}{1-i\Delta_V}\right)(1-i\Delta_H)(1-i\Delta_V)}$$

$$r^{\uparrow/\downarrow}_{V\to V} = 1 - 2\, \eta_{\text{top,V}}\, \frac{\left(1 - i\Delta^{\uparrow/\downarrow}_{QD}\right)(1-i\Delta_H) + C_H}{\left(\left(1 - i\Delta^{\uparrow/\downarrow}_{QD}\right) + \frac{C_H}{1-i\Delta_H} + \frac{C_V}{1-i\Delta_V}\right)(1-i\Delta_H)(1-i\Delta_V)}$$

$$r^{\uparrow/\downarrow}_{H\to V} = \mp 2\, i\, \sqrt{\eta_{\text{top,H}}\, \eta_{\text{top,V}}}\, \frac{\sqrt{C_H\, C_V}}{\left(\left(1 - i\Delta^{\uparrow/\downarrow}_{QD}\right) + \frac{C_H}{1-i\Delta_H} + \frac{C_V}{1-i\Delta_V}\right)(1-i\Delta_H)(1-i\Delta_V)}$$

$$r^{\uparrow/\downarrow}_{H\to H} = 1 - 2\, \eta_{\text{top,H}}\, \frac{\left(1 - i\Delta^{\uparrow/\downarrow}_{QD}\right)(1-i\Delta_V) + C_V}{\left(\left(1 - i\Delta^{\uparrow/\downarrow}_{QD}\right) + \frac{C_H}{1-i\Delta_H} + \frac{C_V}{1-i\Delta_V}\right)(1-i\Delta_H)(1-i\Delta_V)}$$

The semiclassical approximation is also valid when the laser is far-detuned from the emitter, i.e. when the term $\Delta^{\uparrow/\downarrow}_{QD}$ largely dominates compared to the ratios $\frac{C_H}{1-i\Delta_H}$ and $\frac{C_V}{1-i\Delta_V}$. In such a case the reflectivity coefficients are simply given by the empty cavity response, i.e. they become equal to:

$$r^{(\text{cav})}_{V\to V} = 1 - \frac{2\eta_{\text{top,V}}}{1-i\Delta_V} \qquad r^{(\text{cav})}_{V\to H} = 0$$
$$r^{(\text{cav})}_{H\to H} = 1 - \frac{2\eta_{\text{top,H}}}{1-i\Delta_H} \qquad r^{(\text{cav})}_{H\to V} = 0$$

Such equations constitute a generalization of those already used in Arnold et al ([4]), extended to the case of a birefringent cavity, and expressed in the H/V basis. For each ground state $|\uparrow\rangle$, $|\downarrow\rangle$, $|\varnothing\rangle$, one can also deduce the produced polarisation states in the semiclassical regime, through :

$$|\Psi_{\uparrow/\downarrow/\varnothing}\rangle = \frac{b^{\uparrow/\downarrow/\varnothing}_{\text{out},H}\ |H\rangle\ +\ b^{\uparrow/\downarrow/\varnothing}_{\text{out},V}\ |V\rangle}{\sqrt{|b^{\uparrow/\downarrow/\varnothing}_{\text{out},H}|^2 + |b^{\uparrow/\downarrow/\varnothing}_{\text{out},V}|^2}},$$

where the output field amplitudes $b^{\uparrow/\downarrow/\varnothing}_{\text{out},H/V}$ are derived from the above-mentioned reflectivity coefficients.

We note that the above equations are quite general: they are valid for any input polarisation, and for any longitudinal magnetic field (whose main effect is simply to shift the energies $\omega^{\uparrow/\downarrow}_{\text{QD}}$, appearing in $\Delta^{\uparrow/\downarrow}_{\text{QD}}$). At zero magnetic field, for example, $\omega^{\uparrow}_{\text{QD}} = \omega^{\downarrow}_{\text{QD}}$, yet spin-dependant polarisation states are still obtained. This is due to the $\pm$ sign (respectively, $\mp$) appearing in the reflectivity coefficient $r_{V\to H}$ (resp. $r_{H\to V}$), which is positive (resp. negative) in the case of a spin in the $|\uparrow\rangle$ state, and negative (resp. positive) in the case of a spin in $|\downarrow\rangle$. This implies that, at zero field, $r^{\uparrow}_{V\to H} = -r^{\downarrow}_{V\to H}$ while on the contrary $r^{\uparrow}_{V\to V} = r^{\downarrow}_{V\to V}$.

In the present work, when the QD is in the state $|\uparrow\rangle$ the reflectivity coefficients are given by $r^{\uparrow}_{V\to H}$, $r^{\uparrow}_{V\to V}$, $r^{\uparrow}_{H\to V}$ and $r^{\uparrow}_{H\to H}$, while they are given by the empty cavity ones when the system is in state $|\downarrow\rangle$ (since $\Delta^{\downarrow}_{QD}$ remains very large) and when the system is in state $|\varnothing\rangle$. When $|\Psi_{\text{in}}\rangle = |V\rangle$, the expressions further simplify to Eq. (1) of the main text for $|\Psi_{\uparrow}\rangle$, and simplify to $|\Psi_{\downarrow}\rangle = |\Psi_{\varnothing}\rangle = |V\rangle$ for the system states $|\downarrow\rangle$ and $|\varnothing\rangle$.

The above calculations are useful to understand the physics of spin-photon interfaces, thanks to the analytical description of the pure states $|\Psi_{\uparrow/\downarrow/\varnothing}\rangle$, obtained in the linear low-power regime and in absence of fluctuations. We now introduce an independent approach based on numerical simulations performed with the Quantum Optics Toolbox [5]. This consists in numerically solving the master equation in the stationary regime, without any approximation, in order to compute the steady-state density matrix $\hat{\rho}_{\mathrm{ss}}$. This allows deducing the reflected intensities in any polarisation X, with:

$$I_X = \langle \hat{b}^\dagger_{\mathrm{out},X} \hat{b}_{\mathrm{out},X} \rangle = Tr\left(\hat{b}^\dagger_{\mathrm{out},X} \hat{b}_{\mathrm{out},X}\ \hat{\rho}_{\mathrm{ss}} \right)$$

One can also numerically force the system to be initialized in state $|\uparrow\rangle$, $|\downarrow\rangle$, or $|\varnothing\rangle$, and deduce the corresponding conditional intensities :

$$I_X^{(\uparrow/\downarrow/\varnothing)} = \langle \hat{b}^\dagger_{\mathrm{out},X} \hat{b}_{\mathrm{out},X} \rangle_{\uparrow/\downarrow/\varnothing}$$

As a first verification, we use this approach to check that numerical predictions confirm the validity of the analytical formulae, as illustrated in Supplementary Figure 1. In the numerical simulations for this figure, most parameters are kept identical to those in the main text, except that $\sigma_{\mathrm{SF}} = 0$ (no spectral fluctuations) and that the system is numerically forced to stay in the two-level subspace of states $|\uparrow\rangle$ and $|\Uparrow\downarrow\uparrow\rangle$. This allows the absence of fluctuations, which, in addition to the choice of a low-power excitation (so that $P(|\Uparrow\downarrow\uparrow\rangle) \ll 1$), ensures compatibility with the validity domain of the semiclassical approximation. The numerically-simulated intensities (left panel of Supplementary Figure 1) and Stokes parameters (right panel of Supplementary Figure 1) are then strictly superposed to the analytical ones, deduced using the semiclassical reflectivity coefficients and the semiclassical equation $\langle \hat{b}^\dagger_{\mathrm{out},X} \hat{b}_{\mathrm{out},X} \rangle_\uparrow = \left| \langle \hat{b}_{\mathrm{out},X} \rangle_\uparrow \right|^2$.

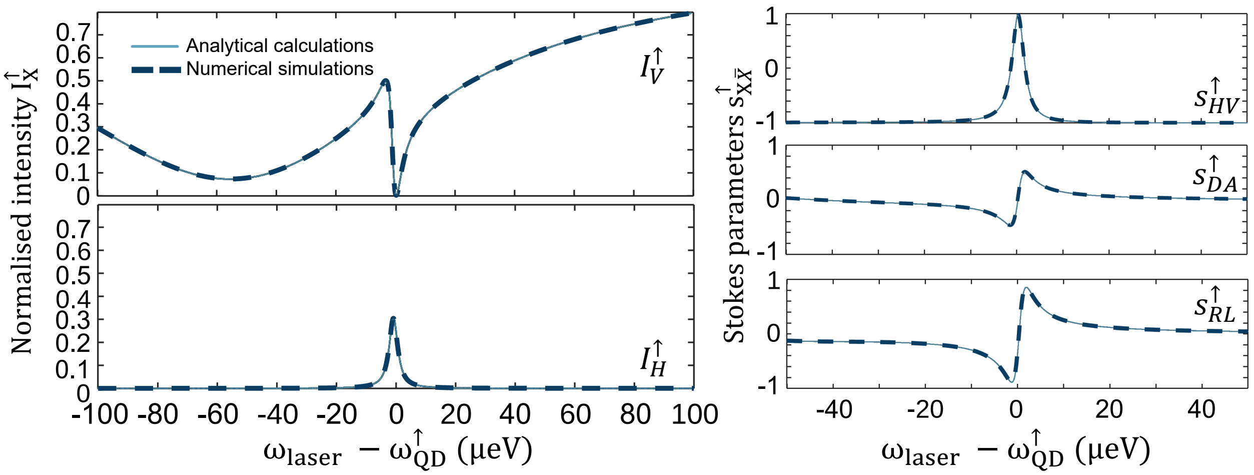

Supplementary Fig. 1. Normalised intensities $I_{H/V}^\uparrow$ (left panel) and the Stokes parameters (right panel) as a function of the detuning between the laser and the energy $\omega_{\mathrm{QD}}^\uparrow$, simulated with the semiclassical model (analytical equations, thin solid lines) and with numerical simulations (dashed lines). The chosen parameters are those from the main text.

The numerical approach is actually more general, as it can provide an exact numerical solving of the master equation, even when the semiclassical approximation fails. We use it to compute time-dependent quantities, to take into account spectral fluctuations, and to fully describe co-tunneling processes: this will be described in Supplementary Note 4. Interestingly, however, such a complete treatment is not required for steady-state calculations of the average intensities, as long as fluctuations are significantly slower than the Purcell-enhanced emission time (around 200 ps). In such a case, one can generally consider that the system is, at each time t, in a well-defined state $|\uparrow\rangle$, $|\downarrow\rangle$, or $|\varnothing\rangle$, and with a fixed value of the transition energies, leading to well-defined reflected intensities $I_X^{(\uparrow/\downarrow/\varnothing)}$. The ergodic theorem then allows us to describe all time-integrated intensities using average values, associated to some probability distribution. One can, in particular:

- average intensities over spectral fluctuations of the relevant energy $\omega_{\mathrm{QD}}^\uparrow$, using a gaussian probability distribution with the standard deviation σ_{SF} introduced in the main text.

- average intensities over the three possible ground states with the respective probabilities $P_\uparrow$, $P_\downarrow$, $P_\varnothing$ introduced in the main text. In general, the average intensity $I_X^{\rm avg}$ depends on $I_X^\uparrow$, $I_X^\downarrow$, $I_X^\varnothing$, through:

$$I_X^{\rm avg} = P_\uparrow\, I_X^\uparrow \; + \; P_\downarrow\, I_X^\downarrow \; + \; P_\varnothing\, I_X^\varnothing,$$

which simplifies to Eq. (2) of the main text when $\omega_{QD}^\downarrow$ remains highly detuned from both $\omega_{QD}^\uparrow$ and $\omega_{\rm laser}$. We note that such equations are valid only for classical probability distributions, i.e. incoherent superpositions of the three possible states, as expected in presence of incoherent relaxation mechanisms (e.g. co-tunneling). In contrast, for a photon interacting with a coherent spin superposition, the system would instead be described by an entangled spin-photon state, after the interaction.

Supplementary Note 3 - Parameter estimation using only intensity measurements

The comparison between theory and experiments allows deducing some of the device parameters with satisfying precision, while leaving a significant uncertainty on others, at least when only intensities and Stokes parameters are used for numerical fits. As described in the main text, separate fits allow independantly deducing all the cavity parameters (i.e. κ_H, κ_V, $\omega_{{\rm cav},H}$, $\omega_{{\rm cav},V}$, $\eta_{{\rm top},H}$ and $\eta_{{\rm top},V}$), from the measured empty-cavity spectra (see Supplementary Note 7 - cavity birefringence). The only remaining parameters governing the reflectivity coefficients are :

- the laser energy $\omega_{\rm laser}$, independantly measured with a High Finessse WS6 wavemeter;
- the transition energy $\omega_{\rm QD}^\uparrow$, governing, and thus deduced from, the frequency at which maximal resonance fluorescence is observed;
- the light-matter coupling g, influencing C_H and C_V;
- the spontaneous emission rate into other modes than the cavity modes, $\gamma_{\rm sp}$, influencing C_H and C_V, as well as the normalized detuning $\Delta_{\rm QD}^\uparrow$.

As a consequence, in the absence of noise, there are only two non-independent parameters, g and γ_{sp}. These parameters govern both the amplitude and width of the cross-polarised resonance fluorescence, i.e. how the normalized intensity $I_H^{\rm avg}$ evolves as a function of $\omega_{\rm laser}$. But they also govern the interference between the extracted resonance fluorescence and the directly-reflected laser, which influences all the other intensities $I_X^{\rm avg}$ with $X = V, D, A, R, L$. It is clearly not possible to fit all the experimental intensities using only g and $\gamma_{\rm sp}$, i.e. a model where the charge occupation probability is unity and where there is no spectral fluctuation whatsoever. As introduced in the main text, additional non-independent parameters have to be introduced, such as a non-unity charge occupation probability P_c and/or a non-zero standard deviation of the transition energy fluctuation, $\sigma_{\rm SF}$.

The set of parameters in the main text, extracted from the numerical simulations, actually corresponds to an intermediate situation where $\sigma_{\rm SF} = 0.5\,\mu$eV, which agrees well with the typical value for hyperfine-induced noise, for an electron in an annealed InGaAs QD [6]. When taking such a value of $\sigma_{\rm SF}$, a good fit is obtained with an occupation probability $P_c = 94\%$, together with $g = 15\,\mu$eV, and $\gamma_{\rm sp} = 0.35\,\mu$eV, as discussed in the main text. Yet, fitting only the intensities is also compatible with other parameter choices, such as:

- the upper bound $P_{c,\rm max} = 1$, associated to a corresponding upper bound $\sigma_{\rm SF,max} = 0.8\,\mu$eV, together with $g = 14.5\,\mu$eV (almost unchanged) and $\gamma_{\rm sp} = 0.35\,\mu$eV (unchanged).
- the lower bound $\sigma_{\rm SF,min} = 0\,\mu$eV, associated to a corresponding lower bound $P_{c,\rm min} = 0.88$, together with $g = 15.5\,\mu$eV (almost unchanged) and $\gamma_{\rm sp} = 0.35\,\mu$eV (unchanged).

Therefore, by focusing on the measured intensities, one can only deduce that $P_c \in [0.88; 1]$, which leads to $P_\uparrow \in [0.44; 0.5]$. To illustrate how this uncertainty impacts the extrapolation process, we display in Supplementary Figure 2 the extrapolated Stokes vector $\vec{S}_\uparrow$, plotted in the Poincaré sphere for a magnetic field of 1.7 T, also for the lower and upper bounds of parameters. For the upper bound ($P_{c,\rm max} = 1$ and $\sigma_{\rm SF,max} = 0.8\,\mu$eV),

the polarisation purity is limited due to the spectral fluctuations, that lead to a Stokes vector $\vec{S}_\uparrow$ inside the Poincaré sphere. For the lower bound ($P_{c,\min} = 0.88$ and $\sigma_{\mathrm{SF,min}} = 0\,\mu$eV), where spectral fluctuations are absent, the extrapolated Stokes vector $\vec{S}_\uparrow$ stays pure, leading to simulated points exactly at the surface of the sphere.

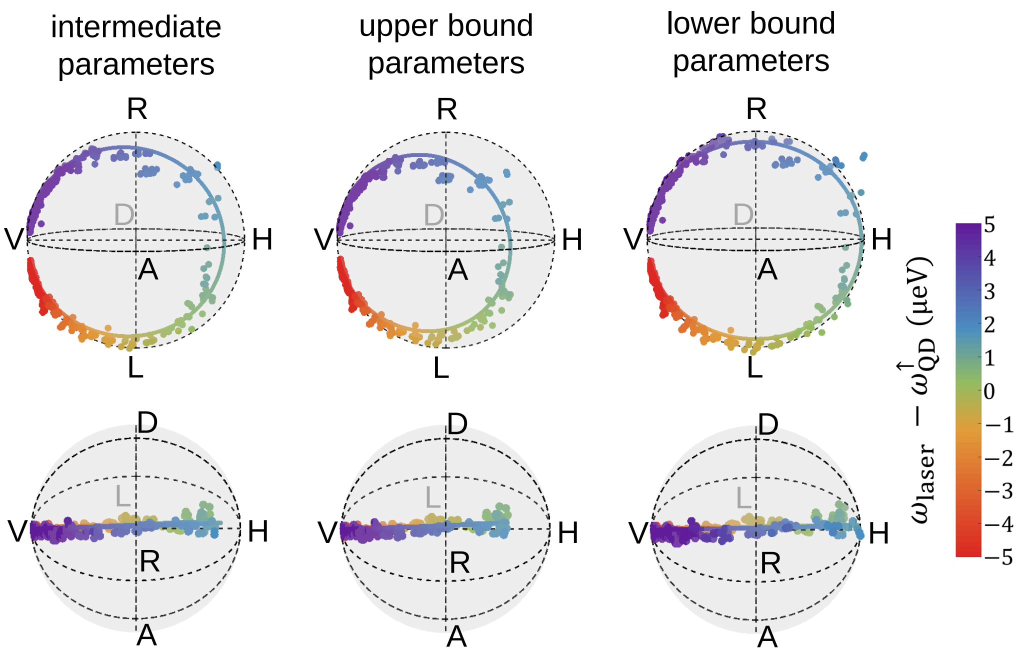

Supplementary Fig. 2. **Extrapolation of the Stokes vector for a pure QD spin state** Extrapolated Stokes vector $\vec{S}_\uparrow$ as a function of $\omega_{\mathrm{laser}} - \omega^{\uparrow}_{\mathrm{QD}}$ (see colorscale) viewed from two different angles in the Poincaré sphere (top/bottom lines) for the intermediate set of parameter used in the main text (left panels), the set of parameters associated to the upper bounds $P_{c,\max} = 1$ and $\sigma_{\mathrm{SF,max}} = 0.8\,\mu$eV (middle panels), and the set of parameters associated to the lower bounds $P_{c,\min} = 0.88$ and $\sigma_{\mathrm{SF,min}} = 0\,\mu$ (right panels). Circles: extrapolation from experimental data, lines: numerical simulations.

If the probability distribution for P_c could be considered rectangular, in the interval $P_c \in [0.88; 1]$, then the associated standard deviation on P_c would be equal to the half-width of the interval divided by $\sqrt{3}$, i.e $\frac{0.06}{\sqrt{3}} \approx 0.035$. Let's note, however, that both of the extremal bounds correspond to unrealistic situations. On the one hand, spectral fluctuations are expected at least in the form of hyperfine interaction, influencing the electron spin. On the other hand, additional data taken as a function of voltage (see Supplementary Note 5) show the presence of co-tunnelling effects, where the occupation probability appears to directly depend on the bias voltage, instead of being constant (as would be the case if co-tunneling were negligible). As mentioned in the main text, and as discussed in Supplementary Note 5, one can actually improve the uncertainty on P_c by taking into account fits of these additional data. The study of such co-tunneling processes, however, requires a theoretical description based on a complete analysis, including time-dependant processes, as described below.

Supplementary Note 4 - Exact numerical simulations including the full system dynamics

We now describe how exact numerical simulations are performed in presence of fluctuations, independently of any approximation. A complete description of the quantum dot-cavity device requires taking into account potential jumps between the two ground states, between the two excited states, as well as escape/capture processes into and from the empty state $|\varnothing\rangle$. Supplementary Figure 3 displays the potential jump processes

with their respective times.

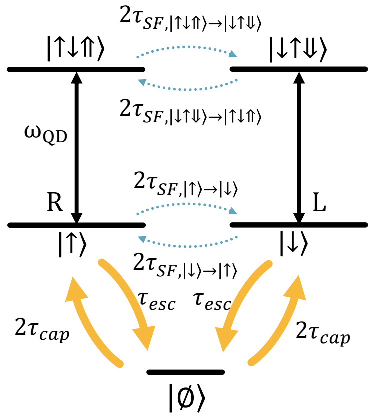

Supplementary Fig. 3. Quantum dot energy levels and potential jumps between states. Escape and capture processes are largely dominant compared to the various spin-flip processes.

As illustrated in this figure, however, only escape and capture processes govern the system's dynamics. Indeed, in our device in the co-tunneling regime, the typical escape time τ_{esc} is at best of several nanoseconds, and the typical capture time of the order of 200 ps (see next Supplementary Note 5). This is by far shorter than all the expected spin-flip times $\tau_{\text{SF},|i\rangle\rightarrow|f\rangle}$, for all initial and final spin states $|i\rangle$ and $|f\rangle$. Indeed, the hyperfine interaction is screened by the applied magnetic field [7]: the lifetime of an electron spin in similar devices would be expected to be well above the millisecond timescale at our high magnetic fields [8], and the expected lifetime of the hole spin is also well above the microsecond timescale, as soon as the applied field reaches a few tens of milliTeslas [9]. Therefore, our numerical simulations are dominated by co-tunneling processes, described through the following collapse operators:

$$\hat{C}_{esc}^{\uparrow/\downarrow} = \frac{1}{\sqrt{\tau_{esc}}} |\varnothing\rangle \langle\uparrow/\downarrow| \quad \text{and} \quad \hat{C}_{cap}^{\uparrow/\downarrow} = \frac{1}{\sqrt{2\tau_{cap}}} |\uparrow/\downarrow\rangle \langle\varnothing| \,.$$

Taking into account these collapse operators, the exact numerical resolution is obtained by finding the averaged density matrix in the steady-state, $\hat{\rho}_{\text{ss}}$. In such steady-state, the value of the occupation probabilities are actually fixed by:

$$P_c = \frac{\tau_{\text{esc}}}{\tau_{\text{esc}} + \tau_{\text{cap}}} \quad \text{and} \quad P_\varnothing = \frac{\tau_{\text{cap}}}{\tau_{\text{esc}} + \tau_{\text{cap}}},$$

together with:

$$P_\uparrow = P_\downarrow = \frac{P_c}{2}.$$

In particular, in this steady-state the equality between $P_\uparrow$ and $P_\downarrow$ is ensured by the rapid successions of escape and capture events: these co-tunneling events each time reset the spin to a random state $|\uparrow\rangle$ or $|\downarrow\rangle$, independently from the (far slower) spin-flip rates, preventing the accumulation of population in any of the spin states.

Interestingly, the exact numerical resolution is also useful to compute transient regimes where, following some initial perturbation, the system's density matrix evolves back to its steady-state value. An important application (see next Supplementary Note) is the calculation of second-order intensity correlations, where a first photon is detected in some polarisation X, later followed by the detection of a second photon, in some polarisation Y [10]. Right after the first detection, the density matrix is projected into the conditional density matrix $\hat{\rho}_X(0^+)$, written as:

$$\hat{\rho}_X(0^+) = \frac{\hat{b}_{\text{out},X} \cdot \hat{\rho}_{ss} \cdot \hat{b}^\dagger_{\text{out},X}}{Tr(\hat{b}_{\text{out},X} \cdot \hat{\rho}_{ss} \cdot \hat{b}^\dagger_{\text{out},X})}.$$

The temporal evolution of this conditional density matrix, $\hat{\rho}_X(t)$, is then deduced by numerically solving the master equation. This allows computing the conditional probability to detect a second photon in polarisation Y after some delay τ, and thus the second-order intensity correlation function $g^{(2)}_{XY}(\tau)$. Such a fonction is normalized to unity at long delays, thanks to the fact that $\hat{\rho}_X(t)$ eventually evolves back to the steady-state density matrix $\hat{\rho}_{ss}$.

Supplementary Note 5 - Co-tunneling regime and bias dependence

Experimental signatures of co-tunneling, where an electron escapes the quantum dot and is replaced by another one from the Fermi sea, can take various forms. This is mostly seen when comparing experiments at different applied voltage bias: indeed, in a regime dominated by co-tunneling, τ_{esc} and τ_{cap} can drastically vary with small changes of the applied bias, contrary to all the other relevant timescales. A first potential consequence is the variation of the charge occupation probability P_c, which can be mostly seen in a varying amplitude of I_H^{avg}, the cross-polarised resonance fluorescence signal. A second potential consequence is the possibility that τ_{esc} becomes comparable with the Purcell-enhanced emission time ($\approx 200\,\text{ps}$). In such a case the semiclassical approximation fails and the resonance fluorescence linewidth is governed, not just by the lifetime of the trion state, but also by that of the unstable ground state. This would mostly result in a varying width of the cross-polarized signal I_H^{avg}, as a function of the bias voltage.

In addition to such indirect signatures, a regime dominated by co-tunneling can also have a direct signature, based on second-order intensity correlations. In such a case, indeed, fast co-tunneling would govern the rate at which the density matrix $\hat{\rho}_X(t)$ (conditioned to a first detection event in polarisation X at time 0) evolves back to $\hat{\rho}_{ss}$, also displaying a strong dependance with the applied voltage.

In our case, all these signatures have been observed and consistently fitted with the complete numerical model described in the Supplementary Note . The left panels of Supplementary Figure 4 display the experimental measurements of the normalised, cross-polarised intensity I_H^{avg}, as a function of the detuning between the laser and $\omega_{\text{QD}}^{\uparrow}$, at 1.7 T, and for different bias voltages. When the applied voltage diverts from its optimal value of $-0.63\,V$, the quantum dot emission I_H^{avg} broadens and diminishes in amplitude, potentially indicating that the fundamental state of the quantum dot is less stable.

Such interpretation is further supported by the right panels of Supplementary Figure 4, which, in the same experimental conditions, display the measured cross-correlation $g^{(2)}_{HV}(\tau)$, as a function of the delay τ between a first photon, detected in polarisation H, and a second one detected in polarisation V. Three regimes can be observed on these cross-correlation measurements:

- a first transient regime, with a peak starting at zero delay with $g^{(2)}_{HV}$ above unity, and rapidly decaying to below-unity values, on the $\sim 200\,ps$ timescale.
- a second transient regime, where the below-unity values of $g^{(2)}_{HV}$ progressively increase to unity,
- finally, at long delays, the stationary regime for which $g^{(2)}_{HV}$ stays equal to unity.

The behaviour of the cross-correlation function $g^{(2)}_{HV}$ will only be briefly commented here, as a complete discussion would go beyond the scope of the present article. After detection of a first H-polarised photon, the conditional density matrix $\hat{\rho}_H(0^+)$ corresponds with certainty to a QD in the $|\Uparrow\rangle$ state. At this instant, there can be no QD-induced dipole, i.e. no resonance fluorescence emission interfering with the laser. Thus, the system simply behaves as an empty cavity, and the probability to detect a second V-polarised photon is proportionnal to the intensity I_V^{cav} (see the corresponding optical response in the middle panel of Fig. 3a in the main text). The first transient regimes then appears at the radiative relaxation timescale: it indeed comes from the fact that a dipole oscillation quickly rebuilds, driven by the CW excitation, which allows recovering an interference between the empty-cavity reflected laser and the QD-induced emission. Ideally, at the end of this first transient regime, the system behaves as a QD-cavity system with a spin perfectly initialized in the $|\Uparrow\rangle$ state, and the probability to detect a second V-polarised photon should be proportionnal to $I_V^{\Uparrow}$ (see right panel of main Fig. 3a). Yet, one also has to take into account the second transient regime, governed by the escape

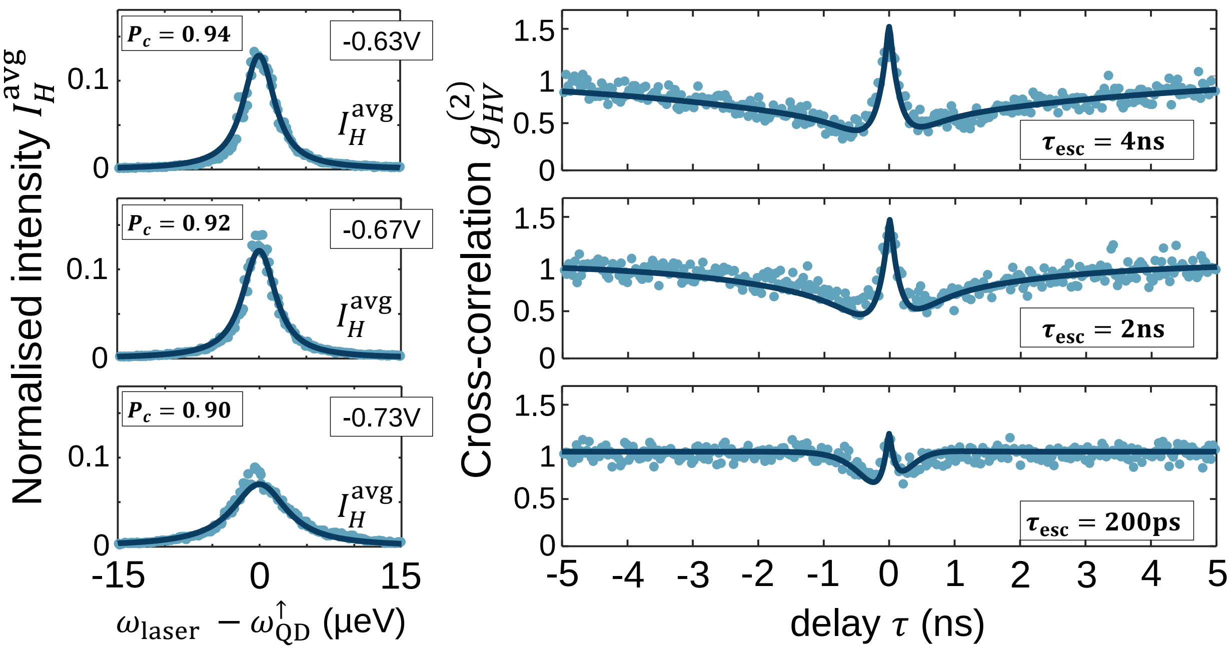

Supplementary Fig. 4. Left panel: normalised intensity I_H^{avg} as a function of the detuning $\omega_{\text{laser}} - \omega_{\text{QD}}^{\uparrow}$ at 1.7 T and for different values of V_{bias}. Right panel: cross-correlation measurements $g_{HV}^{(2)}$ as a function of the delay τ for the corresponding values of V_{bias}. (circles: experimental data, lines: numerical simulations)

time τ_{esc}, which resets the system to a random ground state: the conditional density matrix evolves back to $\hat{\rho}_{ss}$. The probability to detect a second V-polarised photon thus recovers its steady-state value I_V^{avg} (see left panel of main Fig. 3a), which is translated into a second-order cross-correlation evolving back to unity.

In Supplementary Figure 4, the experimental data are superposed with the simulated intensities and cross-correlations. The occupation probability P_c and the escape time τ_{esc} are chosen to fit I_H^{avg} and $g_{HV}^{(2)}(\tau)$, respectively, while the capture time τ_{cap} is deduced with P_c and τ_{esc}. The optimal configuration, with an applied bias of $V_{\text{bias}} = -0.63\,\text{V}$, is best fitted with the parameters given in the main text, additionally providing the information that $\tau_{\text{esc}} = 4\,ns$ (as deduced from the cross-correlation), and $\tau_{\text{cap}} = 250\,ps$, so that $P_c = 0.94$. This is the longest escape time achieved: for slightly different voltages, for example, the escape time is diminished to $\tau_{\text{esc}} = 2\,\text{ns}$ at $-0.67\,\text{V}$, and $\tau_{\text{esc}} = 200\,\text{ps}$ at $-0.73\,V$. This decrease of the escape time also readily explains the broadening of the resonance fluorescence intensity curve, when the ground state becomes unstable at the same timescale as the trion state. In parallel, the occupation probability is slightly modified but keeps large values, thanks to the capture time also shortening to around 170 ps at $-0.67\,\text{V}$ and around 22 ps at $-0.73\,\text{V}$. This behaviour is also expected since both capture and escape rates are governed by tunneling through the same 25 nm barrier, between the quantum dot layer and the n-doped layer (see Methods and Refs. [11, 12] for the sample structure). Note that the standard errors indicated in the main text, i.e. $\sigma_{\text{SF}} = 0.5 \pm 0.2\,\mu\text{eV}$ and $P_c = 0.94 \pm 0.03$, are also deduced considering the fits at the optimal voltage of $-0.63\,\text{V}$: different combinations of σ_{SF} and P_c values fail to reproduce as well the experimental results. These final estimations of σ_{SF} and P_c thus offer more precision compared to the previously-mentioned intervals $P_c \in [0.88; 1]$ and $\sigma_{\text{SF}} \in [0\,\mu\text{eV}; 0.8\,\mu\text{eV}]$, deduced by fitting the intensities alone (as in Supplementary Note 3).

Supplementary Note 6 - Analysis of spin-photon mapping in the limit of large birefringence

In this work, we studied a quantum dot-cavity device with a moderate birefringence, where the two modes have a partial spectral overlap. We limited ourselves to a V-polarised excitation, i.e. $\langle \hat{b}_{\text{in},H}\rangle = 0$, so that the pure output state $|\Psi_\uparrow\rangle$ is given by:

$$|\Psi_\uparrow\rangle \propto r^\uparrow_{V\to H}\,|H\rangle + r^\uparrow_{V\to V}\,|V\rangle\,,$$

In particular, the possibility of full polarisation reversal (π rotation in the Poincaré sphere, as experimentally reported in the main text) is permitted by the possibility to achieve $r^\uparrow_{V\to V} = 0$, while maintaining a potentially large value of the non-diagonal coefficient $r^\uparrow_{V\to H}$.

Here we discuss the limit of a highly-birefringent cavity ($\omega_{\text{cav},H} - \omega_{\text{cav},V} \gg \kappa_{H/V}$), for which the reflection coefficients take a simplified form. To illustrate this, let us consider the case where the laser frequency is chosen in the vicinity of the cavity mode V, i.e. $\Delta_H \gg 1$. Then, H-polarised incoming photons are forbidden to enter the cavity, and are simply reflected in the exact same polarisation H, leading to :

$$r_{H\to H} = 1 \qquad r_{H\to V} = 0$$

Conversely, V-polarised photons are allowed to enter the cavity and excite the emitter. However, there is no Purcell enhancement for emission in the far-detuned H-polarised cavity mode, so that:

$$r_{V\to H} = 0.$$

In such a case, it is impossible to obtain any polarisation rotation by exciting along one of the cavity eigenaxes: exciting the device with V-polarised (resp. H-polarised) light only provides V-polarised (resp. H-polarised) output. Additionnally, there is no possibility to realize any spin-polarisation mapping at zero magnetic field ($\omega^\uparrow_{\text{QD}} = \omega^\downarrow_{\text{QD}}$), since $r^\uparrow_{V\to V} = r^\downarrow_{V\to V}$ in this case, always yielding identical output states : $|\Psi_\uparrow\rangle = |\Psi_\downarrow\rangle$. This is the reason why all spin-photon gates reported with highly-birefringent cavities had to use large magnetic fields [13, 14], and input polarisation states taken as superpositions of the form

$$|\Psi_{\text{in}}\rangle = \alpha\,|H\rangle + \beta\,|V\rangle\,,$$

with $|\alpha|^2 + |\beta|^2 = 1$. This can lead to different polarisation outputs:

$$|\Psi_\uparrow\rangle = \frac{\alpha\,|H\rangle + \beta r^\uparrow_{V\to V}\,|V\rangle}{\sqrt{|\alpha|^2 + |\beta r^\uparrow_{V\to V}|^2}} \qquad \text{and} \qquad |\Psi_\downarrow\rangle = \frac{\alpha\,|H\rangle + \beta r^\downarrow_{V\to V}\,|V\rangle}{\sqrt{|\alpha|^2 + |\beta r^\downarrow_{V\to V}|^2}}.$$

In addition, at high fields, only one of the Zeeman-split transitions (say, at frequency $\omega^\uparrow_{\text{QD}}$) can be in the vicinity of ω_{laser}, so we can also take $\Delta^\downarrow_{\text{QD}} \gg 1$ and work with simplified spin-dependent reflection coefficients :

$$r^\uparrow_{V\to V} = 1 - \frac{2\eta_{\text{top},V}\left(1 - i\Delta^\uparrow_{\text{QD}}\right)}{(1 - i\Delta_V)\left(1 - i\Delta^\uparrow_{\text{QD}}\right) + C_V}$$

$$r^\downarrow_{V\to V} = 1 - \frac{2\eta_{\text{top},V}}{1 - i\Delta_V}$$

From these coefficients, one can find the experimental conditions potentially leading to the orthogonality of the output polarisation states, ie. $\langle\Psi_\uparrow|\Psi_\downarrow\rangle = 0$. Starting from $|\Psi_{\text{in}}\rangle = \alpha\,|H\rangle + \beta\,|V\rangle$, the scalar product between the two possible output states can be written as:

$$\langle\Psi_\uparrow|\Psi_\downarrow\rangle \;=\; \frac{|\alpha|^2 + (1 - |\alpha|^2)(r^\uparrow_{V\to V})^* r^\downarrow_{V\to V}}{\left(\sqrt{|\alpha|^2 + |\beta r^\uparrow_{V\to V}|^2}\right)^* \sqrt{|\alpha|^2 + |\beta r^\downarrow_{V\to V}|^2}}.$$

To find which incoming states $|\Psi_{in}\rangle = \alpha |H\rangle + \beta |V\rangle$ allow reaching $\langle\Psi_\uparrow|\Psi_\downarrow\rangle = 0$, for a given set of $(C_V,\, \eta_{top,V})$, we have computed the minimum of the scalar product, for a broad range of ω_{laser} and $\omega_{QD}^{\uparrow}$, as a function of α. The result is plotted in Supplementary Figure 5, where one observes that the minimal value of $|\langle\Psi_\uparrow|\Psi_\downarrow\rangle|$ stays at zero (i.e. there exist experimental conditions for perfect spin-photon mapping) up to some threshold α_{opt}, after which the minimal value of $|\langle\Psi_\uparrow|\Psi_\downarrow\rangle|$ increases (no perfect spin-photon mapping). We note, in addition, that smaller values of α lead to smaller probabilities for a photon to be reflected when the spin is in the $|\uparrow\rangle$ state. The optimal configuration is thus to work close to the threshold α_{opt}, i.e. the highest value of α allowing perfect mapping:

$$|\alpha_{opt}| = \sqrt{\frac{(1 + C_V - 2\eta_{top,V})(2\eta_{top,V} - 1)}{2\eta_{top,V}(2 + C_V - 2\eta_{top})}},$$

which is generally bounded by $0 \leq |\alpha_{opt}| < \frac{1}{\sqrt{2}}$. Working close to this threshold allows maximizing the denominator of the scalar product $\langle\Psi_\uparrow|\Psi_\downarrow\rangle$, hence the probability for a photon to be actually reflected (either in state $|\Psi_\uparrow\rangle$ or $|\Psi_\downarrow\rangle$).

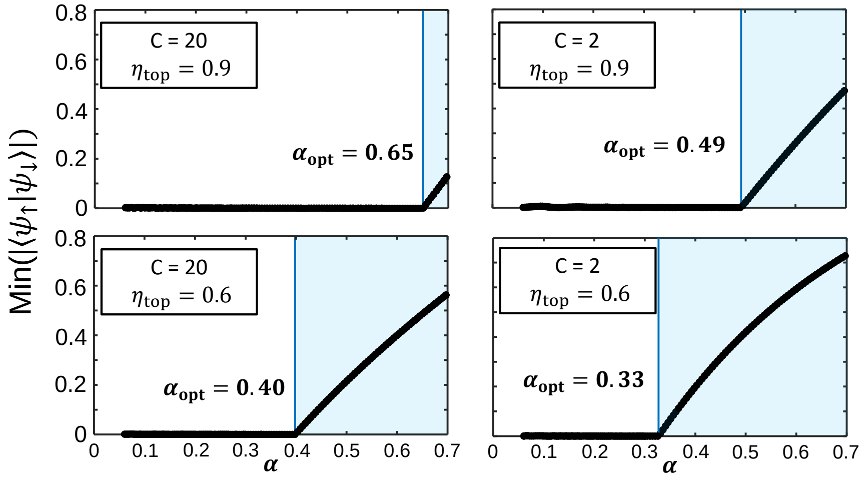

Supplementary Fig. 5. Minimal values of the scalar product $|\langle\Psi_\uparrow|\Psi_\downarrow\rangle|$ as a function of the incoming polarisation state defined by α, for different values of $(C_V,\, \eta_{top,V})$. The maximum value of α allowing two orthogonal output states is limited by α_{opt}, which depends on C_V and $\eta_{top,V}$. The colored areas represent the range of incoming polarisation states where the ideal spin-photon interface condition is not reached.

When choosing an input state at this threshold, $|\alpha| = |\alpha_{opt}|$, the orthogonality of the output states is obtained at full resonance, $\omega_{laser} = \omega_{QD}^{\uparrow} = \omega_{cav,V}$. For $|\alpha| < |\alpha_{opt}|$, the orthogonality of the output states can also be reached, using a laser frequency out of resonance with the cavity mode and the $|\uparrow\rangle - |\uparrow\downarrow\Uparrow\rangle$ transition. As can be seen in Supplementary Figure 5, increasing the device cooperativity and its top-mirror output coupling allows increasing α_{opt}, and thus obtaining a wider range of possible input polarisation states compatible with perfect spin-photon mapping.

In the limit of an ideal device with $C_V \gg 1$ and $\eta_{top,V} = 1$, $r_{V\to V}^{\uparrow} = 1$ while $r_{V\to V}^{\downarrow} = -1$, allowing $|\alpha_{opt}|$ to reach $\frac{1}{\sqrt{2}}$. This is only in this specific limit that one should use a simple input state such as $|D\rangle = \frac{1}{\sqrt{2}}(|H\rangle + |V\rangle)$, to obtain perfect spin photon mapping at resonance with $|\Psi_\uparrow\rangle = |D\rangle$ and $|\Psi_\downarrow\rangle = |A\rangle$, as in the pioneering Duan-Kimple protocol [15]). Interestingly, what we find is that one should *not* use trivial states, such as $|\alpha| = \frac{1}{\sqrt{2}}$, with any imperfect device. Indeed, such a choice would by construction prevent reaching spin-photon mapping, since it would correspond to a choice of $|\alpha|$ higher than the device-limited $|\alpha_{opt}|$.

In conclusion, we see that, at high magnetic fields, tuning the input polarisation with the right value of $|\alpha|$ can allow achieving perfect spin-polarisation mapping even with a highly-birefringent cavity. This comes at the price of a non-trivial input polarisation, and of non-trivial output states, which may explain the lack of experimental demonstrations so far. However, the use of a full polarisation tomography approach, as introduced in

the present paper, is a promising way to implement such schemes quite easily, by allowing the experimentalists to directly visualise the achieved polarisation states in the Poincaré sphere.

Supplementary Note 7 - Extended data

Cavity birefringence

The lift of degeneracy in polarisation, between the two cavity modes, is due to the ellipticity of the micropillar. This is evidenced by measuring the cavity reflectivity under vertically- and horizontally-polarised excitation. A V-polarised (or H-polarised) CW laser is sent to the micropillar, while forcing the absence of charge in the QD (applied voltage of 0V). Its wavelength is scanned around the cavity wavelength while the reflected light is measured by a single photon detector. Far from the cavity resonance, the light is completely reflected and the reflectivity goes to unity. At resonance with the cavity, there is a dip in the reflectivity spectrum for each cavity mode as shown in Supplementary Figure 6. We deduce from these reflectivity spectra the energies of the two cavity modes $\omega_{\mathrm{cav},V}$ and $\omega_{\mathrm{cav},H}$, and their damping rates κ_V and κ_H, governing the width of the reflectivity spectra. The contrast of the dip gives their associated effective top mirror output coupling $\eta_{\mathrm{top,eff},V}$ and $\eta_{\mathrm{top,eff},H}$. All these parameters are summarized in Supplementary Table 1. We note that the optical alignment, and in particular the coupling of the reflected beam into the collection fiber, can lead to slight variations of $\eta_{\mathrm{top,eff},V}$ and $\eta_{\mathrm{top,eff},H}$ with respect to $\eta_{\mathrm{top},V}$ and $\eta_{\mathrm{top},H}$. This is made possible by the fact that the collection fiber may not identically collect the light directly reflected by the top mirror and the light escaping from the cavity mode, slightly modifying the interference of the two contributions within the input-output equations $\hat{b}_{\mathrm{out},H/V} = \hat{b}_{\mathrm{in},H/V} + \sqrt{\kappa_{\mathrm{top},H/V}}\,\hat{a}_{H/V}$. This is why, in all our numerical simulations, we actually use $\kappa_{\mathrm{top,eff},H/V}$. The actual top-mirror output coupling efficiencies, and thus the actual values of $\kappa_{\mathrm{top},H/V}$, would be truly accessed only if a perfect mode matching could be achieved, and/or by using free-space polarisation tomography techniques as in Ref. [16].

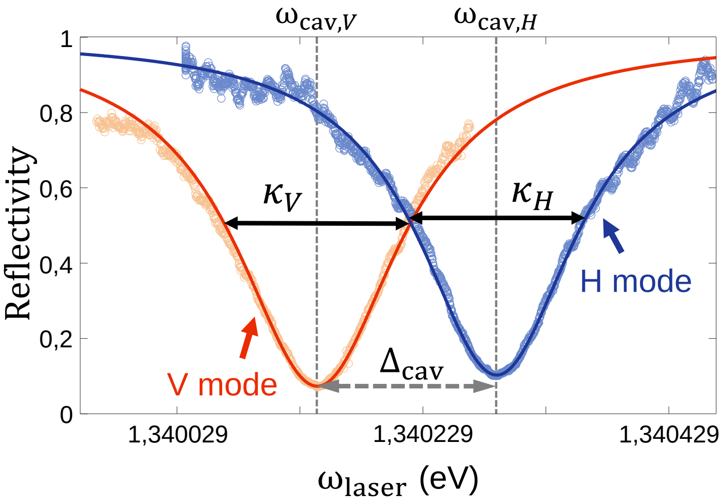

Supplementary Fig. 6. **Reflectivity spectra of the two empty cavity modes.** The cavity has two different modes due to the micropillar ellipticity (circles: experimental data; lines: numerical simulations). The dashed vertical lines indicate the energies of the two cavity modes $\omega_{\mathrm{cav},V}$ and $\omega_{\mathrm{cav},H}$.

V MODE	H MODE
$\omega_{\mathrm{cav},V} = 1,3401430 \pm 0.5 \times 10^{-6}\ eV$	$\omega_{cav,H} = 1,3402890 \pm 0.5 \times 10^{-6}\ eV$
$\kappa_V = 162 \pm 6\ \mu eV$	$\kappa_H = 155 \pm 6\ \mu eV$
$\eta_{\mathrm{top,eff},V} = 0.635 \pm 0.005$	$\eta_{\mathrm{top,eff},H} = 0.660 \pm 0.005$

Supplementary Table 1. Cavity parameters

Residual cavity-induced polarisation rotations

The reflection coefficients of the cavity depend on the cavity modes due to the ellipticity of the micropillar. When the cavity is not strictly excited along its cavity eigenaxis, the reflected light undergoes a cavity-induced polarisation rotation. Experimentally, we thus need to estimate this phenomenon, and minimize it, to ensure that the excitation polarisation is aligned to the cavity mode. This is performed using both a half-waveplate and a quarter-waveplate in the excitation path, to precisely control the incoming polarisation.

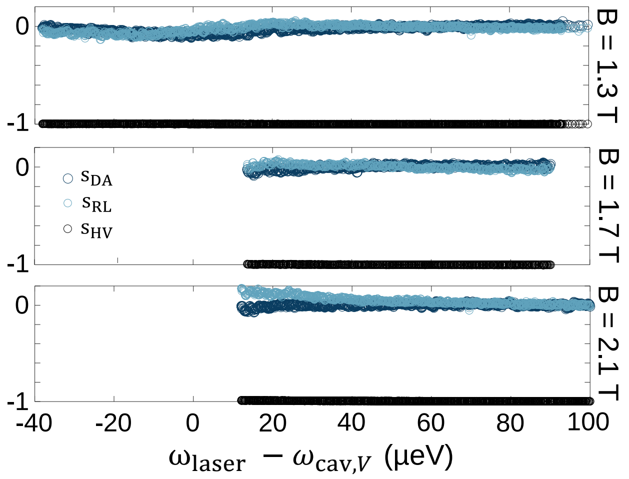

Supplementary Fig. 7. **Cavity axis V excitation** Stokes parameters of the empty cavity as a function of the detuning between the laser and the cavity mode V for three different magnetic fields.

In Supplementary Figure 7, we display the Stokes parameters $s^{\mathrm{cav}}_{X\bar{X}}$ in every polarisation basis $X\bar{X} = HV$, DA and RL for the empty cavity as a function of the energy detuning between the laser and the cavity mode V. Such a characterization is performed for every magnetic field, since slight changes in the experimental conditions may have arised in between successive measurements.

If the excitation polarisation matches the cavity mode V, there is no polarisation rotation induced by the cavity and the reflected light is always V-polarised. In this case, $s^{\mathrm{cav}}_{HV} = -1$ and $s^{\mathrm{cav}}_{DA} = s^{\mathrm{cav}}_{RL} = 0$ for every detunings. In Supplementary Figure 7 we see that $s^{\mathrm{cav}}_{HV} = -1$ is indeed well verified for all experiments. The values of s^{cav}_{DA} and s^{cav}_{RL} also remain approximately constant and close to zero, though with small deviations corresponding to unwanted cavity-induced rotations by a few degrees (either in longitude, thus impacting s^{cav}_{DA}, or in latitude, impacting s^{cav}_{RL}). These undesired rotations are noticed in particular at $1.3\,T$, and at $2.1\,T$, especially at the vicinity of the V cavity mode resonance: this corresponds to the energy of the reflectivity dip where the measurement is more sensitive to slight polarisation rotations. We believe these cavity-induced rotations, though being small, participate to the overall experimental error induced on the measurement of the Stokes vector in Figure 2 (see main text), and to the unphysical extrapolated points outside the Poincaré sphere.

Measurements of average and extrapolated Stokes parameters

In Supplementary Figure 8, the measured Stokes parameters $s^{\text{avg}}_{X\bar{X}}$ are displayed as a function of the detuning $\omega_{\text{laser}} - \omega^{\uparrow}_{\text{QD}}$ for the three different magnetic fields. The measurements are performed without initialising the QD state. Experimental data for all the magnetic fields are in satisfying agreement with the numerical simulations. The data from the middle panel at $1.7\,T$ were also presented in Fig. 2b. At $1.3\,T$, the Stokes parameters predicted by the simulation don't fit the experimental data for all detunings, due to cavity-induced polarisation rotation that is not completely eliminated as explained above.

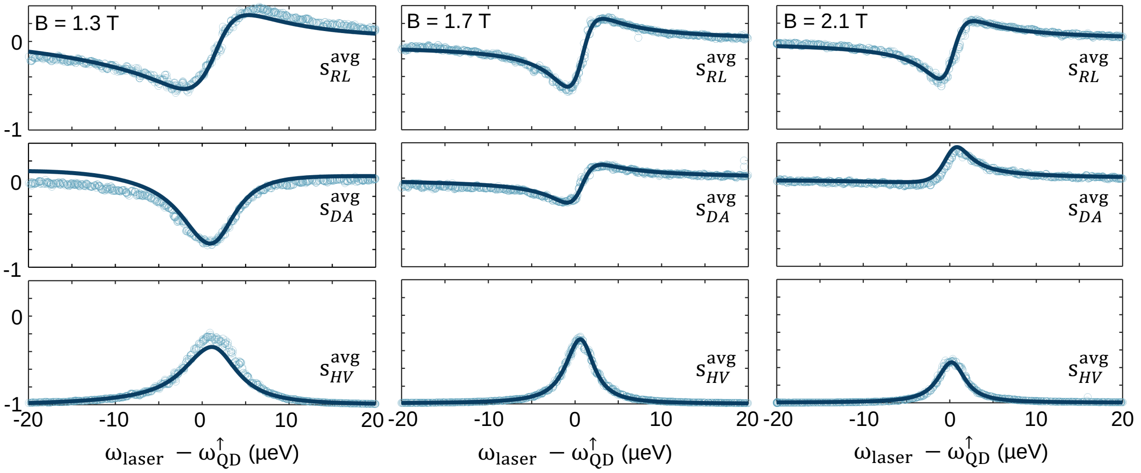

Supplementary Fig. 8. **Polarisation measurement for a fluctuating QD state**. Measured Stokes parameters $s^{\text{avg}}_{X\bar{X}}$ (with $X\bar{X}$ = HV, DA and RL) as a function of the energy detuning between the laser and the transition $|\Uparrow\rangle - |\Uparrow\Downarrow\Uparrow\rangle$ for three different magnetic fields.

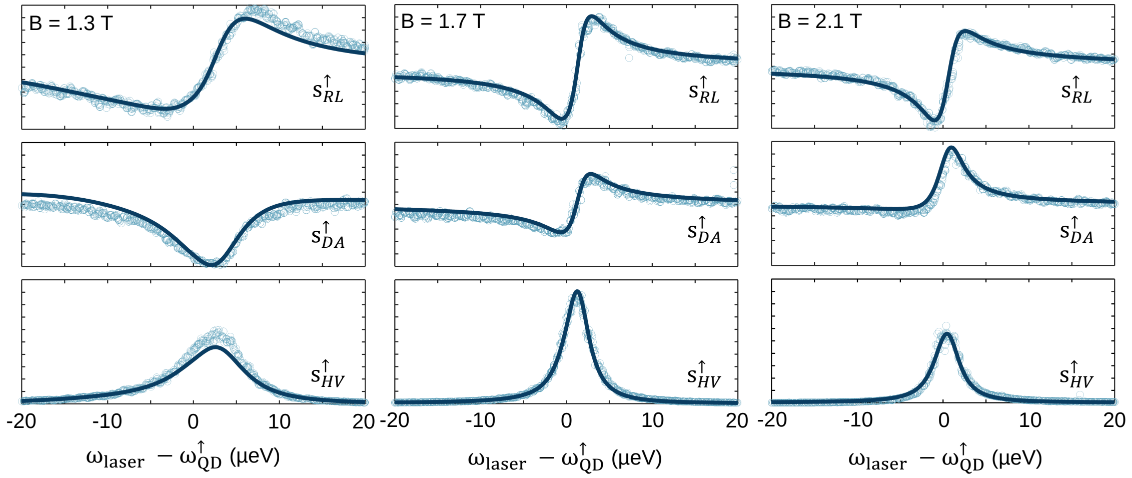

Supplementary Fig. 9. **Generating different output states** Extrapolated Stokes parameters $s^{\uparrow}_{X\bar{X}}$ (with $X\bar{X}$ = HV, DA and RL) as a function of the energy detuning between the laser and the transition $|\Uparrow\rangle - |\Uparrow\Downarrow\Uparrow\rangle$ for three different magnetic fields.

Finally, the extrapolated Stokes parameters $s^{\uparrow}_{X\bar{X}}$ for $X\bar{X}$= HV, DA and RL polarisation bases are plotted in the Supplementary Figure 9 as a function of the detuning between the laser and the $|\Uparrow\rangle - |\Uparrow\Downarrow\Uparrow\rangle$ transition energy for the three magnetic fields. At $1.3\,T$, the Stokes parameter $s^{\uparrow}_{DA}$ approaches -1 at resonance, meaning that the output polarisation approaches state $|A\rangle$. At 1.7T, $s^{\uparrow}_{HV}$ approaches $+1$, so the output polarisation

approaches state $|H\rangle$. At $2.1\,T$, $s^{\uparrow}_{DA}$ approaches $+1$, meaning that the output polarisation approaches state $|D\rangle$. In addition, the extrapolated Stokes parameters are in good agreement with the results of the numerical simulation, especially at 1.7T and $2.1\,T$. At $1.3\,T$, there is a difference between the extrapolated data and the prediction from the numerical simulation. This difference is already present in Supplementary Figure 8 and is amplified by the extrapolation process, leading, as previously discussed, to the unphysical points with a polarisation purity slightly above unity.

Simulations of the possible output polarisation states

Fig. 4b of the main text displays how the theoretical output polarisation Stokes vector $\vec{\boldsymbol{S}}_{\uparrow}$ varies in the Poincaré sphere, for various detunings and magnetic fields. The associated dataset is displayed in Supplementary Figure 10, which shows the simulated Stokes components $s^{\uparrow}_{X\bar{X}}$ for $X\bar{X} = HV$, DA and RL, as a function of the detuning between the laser and the V cavity mode energy, for all the magnetic fields considered in Fig. 4b. Full lines consider the exact same parameters as those used in the main text, including in particular spectral fluctuations with standard deviation $\sigma_{\mathrm{SF}} = 0.5\,\mu$eV, while dashed lines consider the same parameters yet without spectral fluctuations. Interesting points can be seen in particular:

- At 1.35 T, where a detuning exists for which $s^{\uparrow}_{DA}$ is equal to -1 without fluctuations (ideal target state $|A\rangle$). With spectral fluctuations it becomes equal to -0.98, implying a fidelity of 99% to state $|A\rangle$.
- At 1.69 T, where a detuning exists for which $s^{\uparrow}_{HV}$ is equal to 1 without fluctuations (ideal target state $|H\rangle$). With spectral fluctuations it becomes equal to 0.81, implying a fidelity of 90.5% to state $|H\rangle$.
- At 2.18 T, where a detuning exists for which $s^{\uparrow}_{DA}$ is equal to 1 without fluctuations (ideal target state $|D\rangle$). With spectral fluctuations it becomes equal to 0.88, implying a fidelity of 94% to state $|D\rangle$.

Moreover, it can be seen in Supplementary Figure 10 that at 1.5 T, a detuning exists for which the Stokes coefficient $s^{\uparrow}_{RL}$ is almost equal to 1 without fluctuations (ideal target state $|R\rangle$). With spectral fluctuations it becomes equal to 0.96, implying a fidelity of 98% to state $|R\rangle$.

An additional information can be found by extending such simulations to all possible values of ω_{laser} and $\omega^{\uparrow}_{\mathrm{QD}}$, to see which part of the Poincaré sphere can experimentally be accessed with a given device, starting from with the single trivial input state $|\Psi_{\mathrm{in}}\rangle = |V\rangle$. As before, in absence of spectral fluctuations, the output pure state $|\Psi_{\uparrow}\rangle$ is given by

$$|\Psi_{\uparrow}\rangle \propto r^{\uparrow}_{V\to H}\,|H\rangle + r^{\uparrow}_{V\to V}\,|V\rangle\,,$$

and the Stokes components of $|\Psi_{\uparrow}\rangle$, can also be expressed as a function of the reflection coefficients:

$$s^{\uparrow}_{HV} = \frac{|r^{\uparrow}_{V\to H}|^2 - |r^{\uparrow}_{V\to V}|^2}{|r^{\uparrow}_{V\to H}|^2 + |r^{\uparrow}_{V\to V}|^2} \qquad s^{\uparrow}_{DA} = \frac{2Re(r^{\uparrow}_{V\to H}\cdot r^{\uparrow *}_{V\to V})}{|r^{\uparrow}_{V\to H}|^2 + |r^{\uparrow}_{V\to V}|^2} \qquad s^{\uparrow}_{RL} = \frac{2Im(r^{\uparrow}_{V\to H}\cdot r^{\uparrow *}_{V\to V})}{|r^{\uparrow}_{V\to H}|^2 + |r^{\uparrow}_{V\to V}|^2}$$

In Supplementary Figure 11, we plot all possible output polarisation states (orange points) in the Poincaré sphere. The computation is performed by aggregating simulations for a broad and dense set of energies ω_{laser} and $\omega^{\uparrow}_{\mathrm{QD}}$, going from very red-detuned ($1.339\,eV$) to very blue-detuned ($1.343\,eV$). The quantum dot and cavity parameters correspond to the device studied in this work, yet without spectral fluctuations. The two plots represent two opposite views of the same sphere.

This aggregation of all the possible states $|\Psi_{\uparrow}\rangle$ allows visualising the polarisation states that can be reached (orange region covering most of the surface), as compared to those that cannot be reached (black region).The delimitation of such a forbidden region depends on all device parameters and its study goes beyond the scope of our paper. Yet, one can directly see that this forbidden region corresponds to specific combinations of longitude/latitude, only in a single "quadrant" (positive rotations in longitude combined with some negative rotations in latitude, compared to the input state $|V\rangle$), and with limited angles. In particular, it does not include any of the directly useful states that one can wish to realize: π rotation (state $|H\rangle$) is directly useful at high magnetic field, $\frac{\pi}{2}$ rotations (all states on the DRAL meridian) can be used at zero field, while all

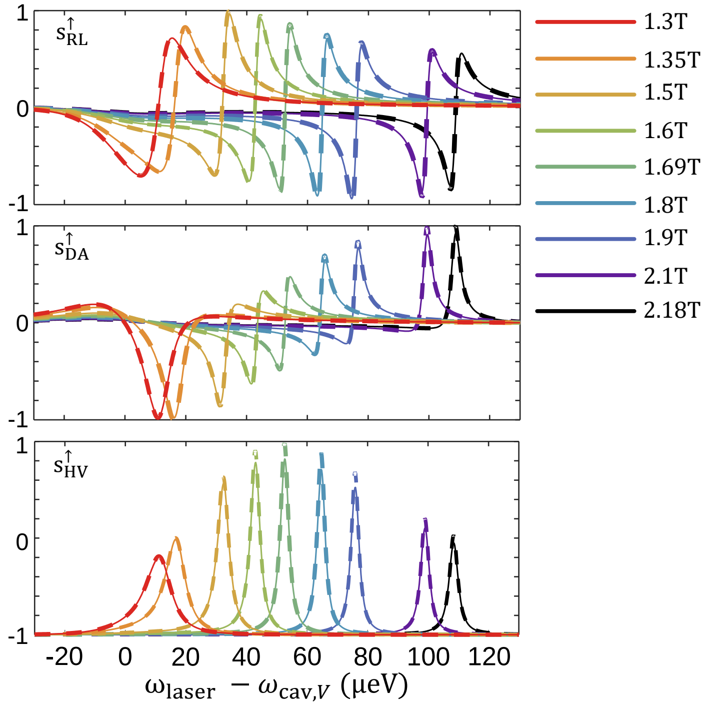

Supplementary Fig. 10. Simulated Stokes parameters $s^{\uparrow}_{X\bar{X}}$ (with $X\bar{X}$ = HV, DA and RL) as a function of the energy detuning between the laser and $\omega_{\text{cav},V}$, for different values of the magnetic field, both with (full lines) and without (dashed lines) spectral fluctuations.

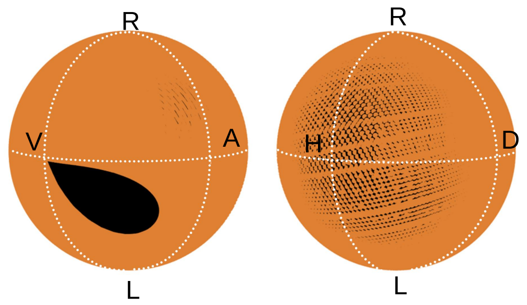

Supplementary Fig. 11. Points on the Poincaré sphere reached by $|\Psi_{\text{out}}\rangle$ calculated in the semi-classical regime with the parameters of the device studied in this work. The trajectories of $|\Psi_{\text{out}}\rangle$ in the Poincaré sphere are calculated for a range of $\omega^{\uparrow}_{\text{QD}}$ from $1.339\,eV$ to $1.343\,eV$, and aggregated in the same sphere.

intermediate rotations between $\frac{\pi}{2}$ and π are available to use for intermediate fields. Overall, most of the polarisation states of the Poincaré sphere can be reached with our device.

---

[1] Walls, D.F. and Milburn, Gerard J., Quantum Optics (Springer, 2008) Chap. 7.

[2] A. Auffèves-Garnier, C. Simon, J.-M. Gérard, and J.-P. Poizat, Giant optical nonlinearity induced by a single two-level system interacting with a cavity in the Purcell regime, Physical Review A **75**, 053823 (2007).

[3] H. S. Nguyen, G. Sallen, C. Voisin, P. Roussignol, C. Diederichs, and G. Cassabois, Ultra-coherent single photon source, Applied Physics Letters **99**, 261904 (2011).

[4] C. Arnold, J. Demory, V. Loo, A. Lemaître, I. Sagnes, M. Glazov, O. Krebs, P. Voisin, P. Senellart, and L. Lanco, Macroscopic rotation of photon polarization induced by a single spin, Nature Commun. **6**, 6236 (2015).

[5] S. M. Tan, A computational toolbox for quantum and atomic optics, J. Opt. B: Quantum Semiclass. Opt **1**, 424 (1999).

[6] E. A. Zhukov, E. Kirstein, D. S. Smirnov, D. R. Yakovlev, M. M. Glazov, D. Reuter, A. D. Wieck, M. Bayer, and A. Greilich, Spin inertia of resident and photoexcited carriers in singly charged quantum dots, Phys. Rev. B **98**, 121304 (2018).

[7] B. Urbaszek, X. Marie, T. Amand, O. Krebs, P. Voisin, P. Maletinsky, A. Högele, and A. Imamoglu, Nuclear spin physics in quantum dots: An optical investigation, Rev. Mod. Phys **7**, 79 (2013).

[8] C.-Y. Lu, Y. Zhao, A. N. Vamivakas, C. Matthiesen, S. Fält, A. Badolato, and M. Atatüre, Direct measurement of spin dynamics in InAs/GaAs quantum dots using time-resolved resonance fluorescence, Physical Review B **81**, 035332 (2010).

[9] R. Dahbashi, J. Hübner, F. Berski, K. Pierz, and M. Oestreich, Optical spin noise of a single hole spin localized in an (inga)as quantum dot, Phys. Rev. Lett. **112**, 156601 (2014).

[10] N. Coste, M. Gundin, D. A. Fioretto, S. E. Thomas, C. Millet, E. Mehdi, N. Somaschi, M. Morassi, M. Pont, A. Lemaître, N. Belabas, O. Krebs, L. Lanco, and P. Senellart, Probing the dynamics and coherence of a semiconductor hole spin via acoustic phonon-assisted excitation, Quantum Science and Technology **8**, 025021 (2023).

[11] N. Somaschi, V. Giesz, L. De Santis, J. C. Loredo, M. P. Almeida, G. Hornecker, S. L. Portalupi, T. Grange, C. Antón, J. Demory, C. Gómez, I. Sagnes, N. D. Lanzillotti-Kimura, A. Lemaítre, A. Auffeves, A. G. White, L. Lanco, and P. Senellart, Near-optimal single photon sources in the solid state, Nature Photon. **10**, 340 (2016).

[12] L. De Santis, C. Antón, B. Reznychenko, N. Somaschi, G. Coppola, J. Senellart, C. Gómez, A. Lemaître, I. Sagnes, A. G. White, L. Lanco, A. Auffèves, and P. Senellart, A solid-state single-photon filter, Nature Nano. **12**, 663 (2017).

[13] H. Kim, R. Bose, T. C. Shen, G. S. Solomon, and E. Waks, A quantum logic gate between a solid-state quantum bit and a photon, Nature Photon. **7**, 373 (2013).

[14] T. G. Tiecke, J. D. Thompson, N. P. de Leon, L. R. Liu, V. Vuletić, and M. D. Lukin, Nanophotonic quantum phase switch with a single atom, Nature **508**, 241 (2014).

[15] L.-M. Duan and H. J. Kimble, Scalable Photonic Quantum Computation through Cavity-Assisted Interactions, Phys. Rev. Lett. **92**, 127902 (2004).

[16] P. Hilaire, C. Antón, C. Kessler, A. Lemaître, I. Sagnes, N. Somaschi, P. Senellart, and L. Lanco, Accurate measurement of a 96% input coupling into a cavity using polarization tomography, Appl. Phys. Lett. **112**, 201101 (2018).
