# Supplementary material for: Giant optical polarisation rotations induced by a single quantum dot spin
Source: Nat Commun. 2024 Jan 18;15:598. doi: 10.1038/s41467-023-44651-8 (PMC10796934; doi:10.1038/s41467-023-44651-8)
Supplement: Supplementary file 3 — Description of Additional Supplementary Files [file 41467_2023_44651_MOESM3_ESM.pdf]

Description of Additional Supplementary Files

Supplementary Movie 1 - Rotation of the average output polarisation in the Poincaré sphere as a function of the detuning (see colorscale) between the laser and the considered quantum dot transition at 1.3T, in the case of the non-initialised quantum dot. This video corresponds to Figure 2c.

Supplementary Movie 2 - Rotation of the average output polarisation in the Poincaré sphere as a function of the detuning (see colorscale) between the laser and the considered quantum dot transition at 1.7T, in the case of the non-initialised quantum dot. This video corresponds to Figure 2c.

Supplementary Movie 3 - Rotation of the average output polarisation in the Poincaré sphere as a function of the detuning (see colorscale) between the laser and the considered quantum dot transition at 2.1T, in the case of the non-initialised quantum dot. This video corresponds to Figure 2c.

Supplementary Movie 4 - Rotation of the extrapolated output polarisation in the Poincaré sphere as a function of the detuning (see colorscale) between the laser and the considered quantum dot transition. This video corresponds to Figure 3b.

Supplementary Movie 5 - Rotation of the extrapolated output polarisation in the Poincaré sphere as a function of the polarisation purity (see colorscale) at 1.3T. The trajectory of the output polarisation in the sphere corresponds however to a scan of the detuning between the laser and the considered quantum dot transition. This video corresponds to Figure 4a.

Supplementary Movie 6 - Rotation of the extrapolated output polarisation in the Poincaré sphere as a function of the polarisation purity (see colorscale) at 1.7T. The trajectory of the output polarisation in the sphere corresponds however to a scan of the detuning between the laser and the considered quantum dot transition. This video corresponds to Figure 4a.

Supplementary Movie 7 - Rotation of the extrapolated output polarisation in the Poincaré sphere as a function of the polarisation purity (see colorscale) at 2.1T. The trajectory of the output polarisation in the sphere corresponds to a scan of the detuning between the laser and the considered quantum dot transition. This video corresponds to Figure 4a.

Supplementary Movie 8 - Numerical simulations of the output polarisation in the Poincaré sphere at different magnetic fields (colors corresponding to the colors of Supplementary Fig. 10), with environmental noise, in the case of the spin initialised. The trajectory of the output polarisation in the sphere corresponds to a scan of the detuning between the laser and the considered quantum dot transition. This video corresponds to Figure 4b.

Supplementary Movie 9 - Numerical simulations of the output polarisation in the Poincaré sphere at different magnetic fields (colors corresponding to the colors of Supplementary Fig. 10), without environmental noise, in the case of the spin initialised. The trajectory of the output polarisation in the sphere corresponds to a scan of the detuning between the laser and the considered quantum dot transition. This video corresponds to Figure 4b.
