# Supplementary figures and images for: Giant optical polarisation rotations induced by a single quantum dot spin
Source: Nat Commun. 2024 Jan 18;15:598. doi: 10.1038/s41467-023-44651-8 (PMC10796934; doi:10.1038/s41467-023-44651-8)

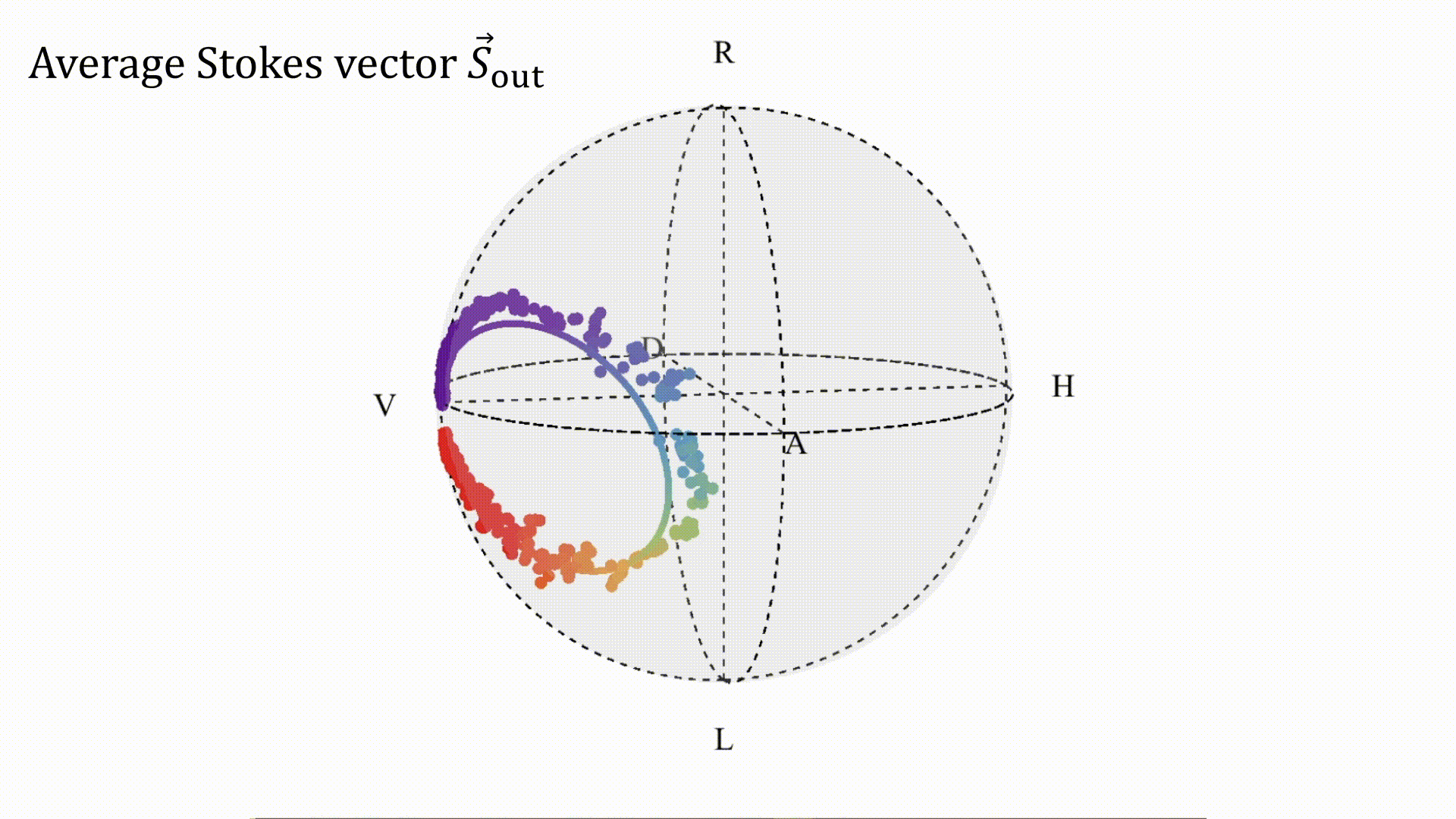

Supplement: Supplementary file 4 — Supplementary Movie 1 [file 41467_2023_44651_MOESM4_ESM.gif]

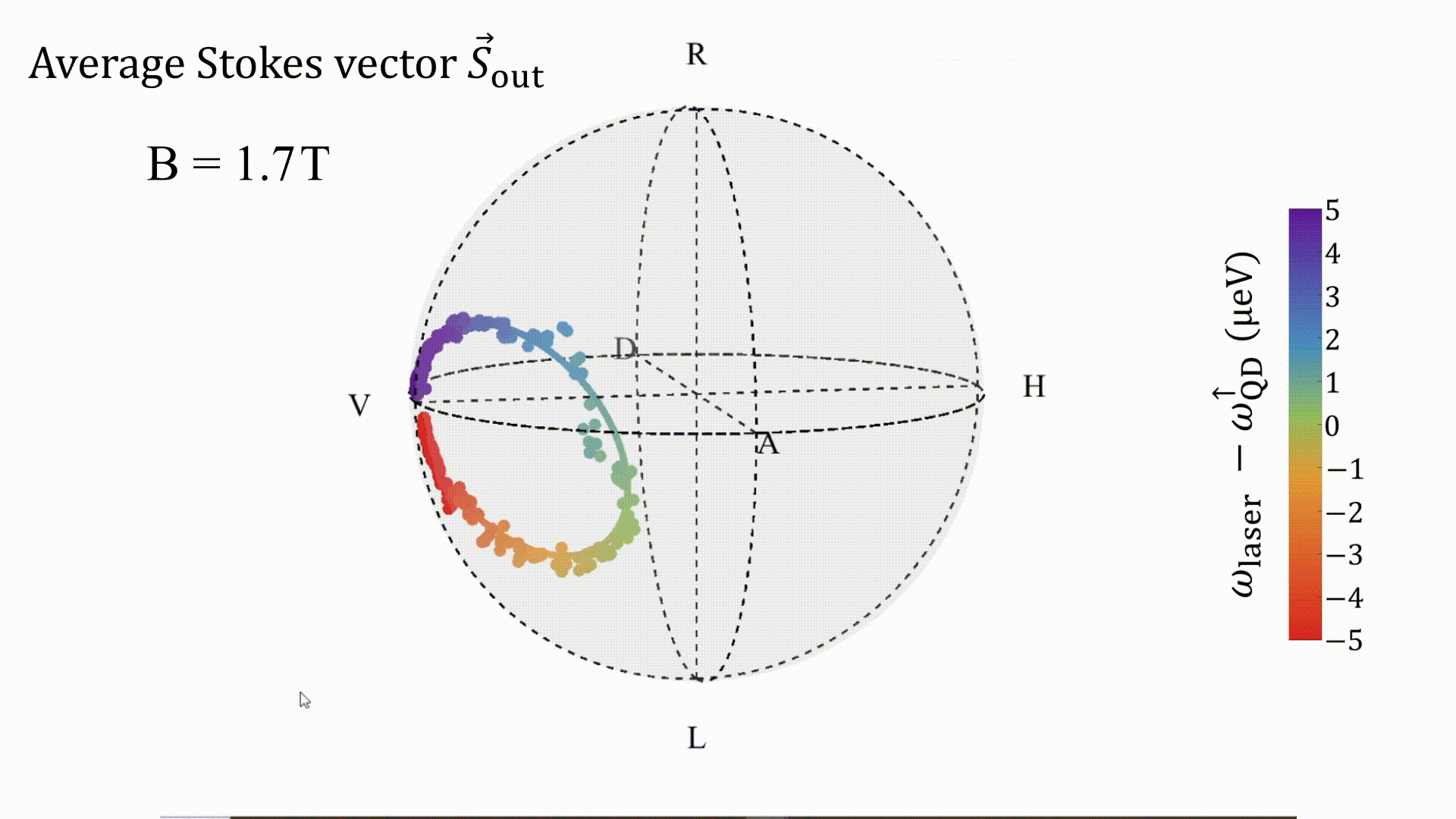

Supplement: Supplementary file 5 — Supplementary Movie 2 [file 41467_2023_44651_MOESM5_ESM.gif]

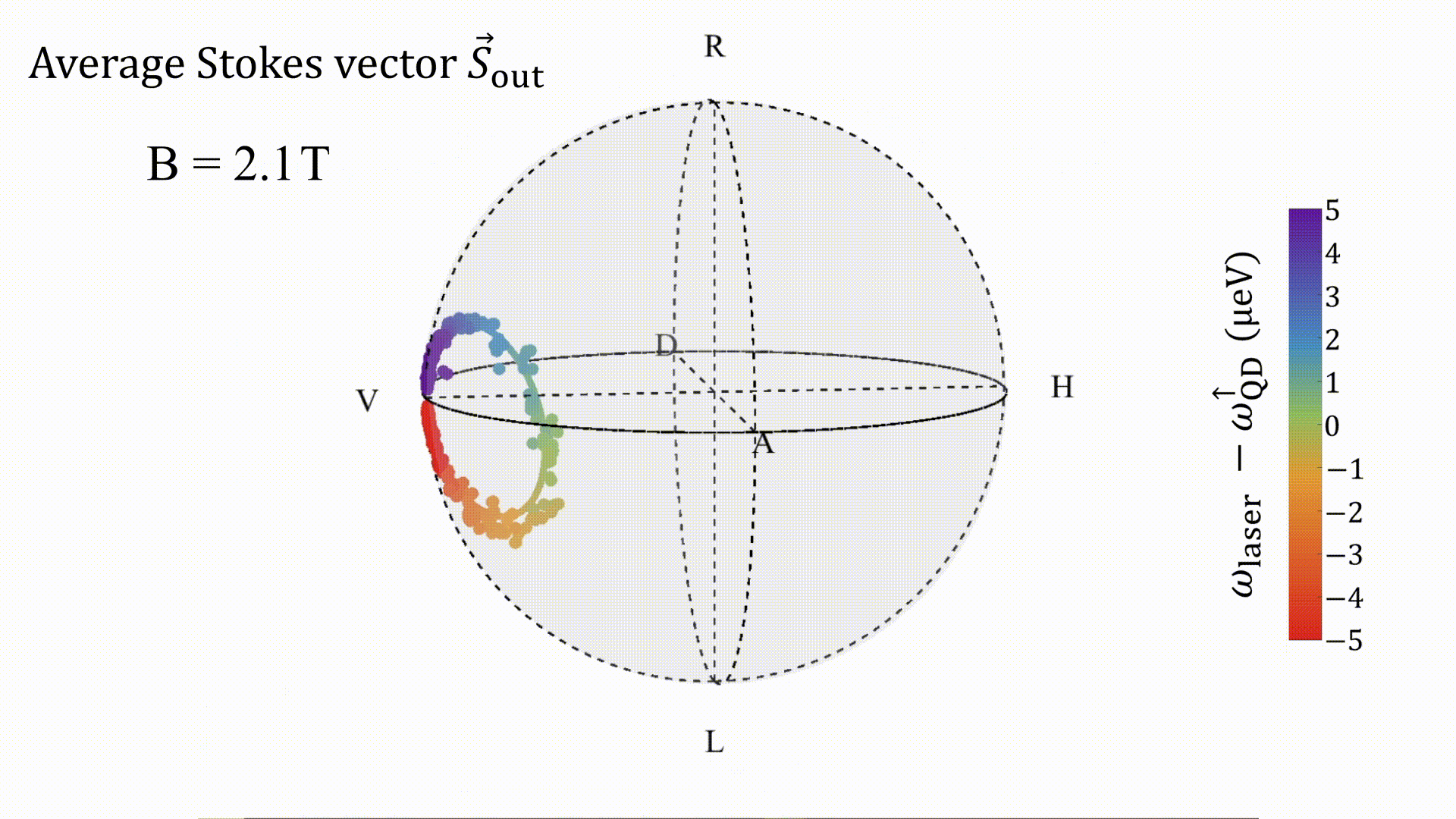

Supplement: Supplementary file 6 — Supplementary Movie 3 [file 41467_2023_44651_MOESM6_ESM.gif]

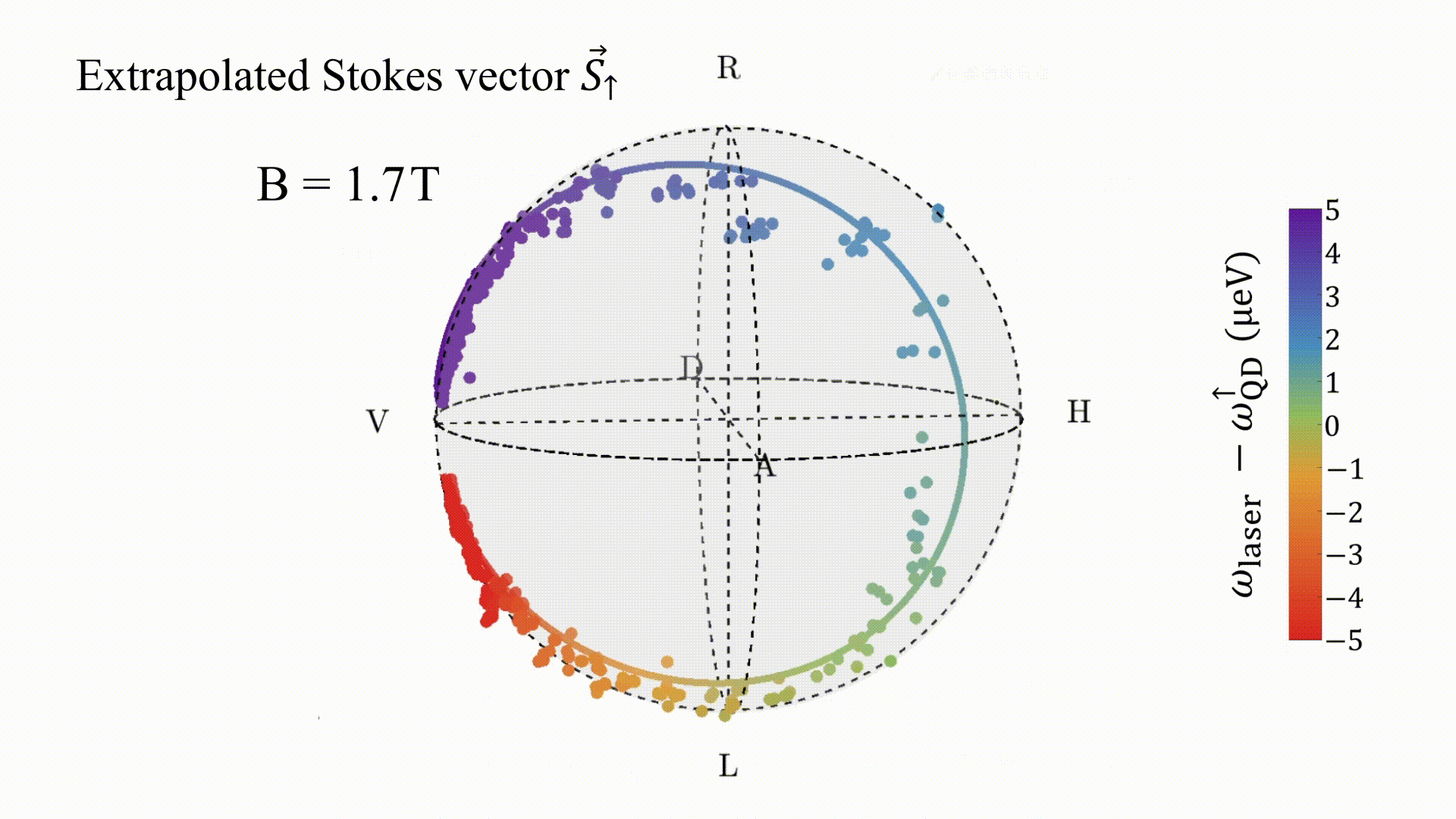

Supplement: Supplementary file 7 — Supplementary Movie 4 [file 41467_2023_44651_MOESM7_ESM.gif]

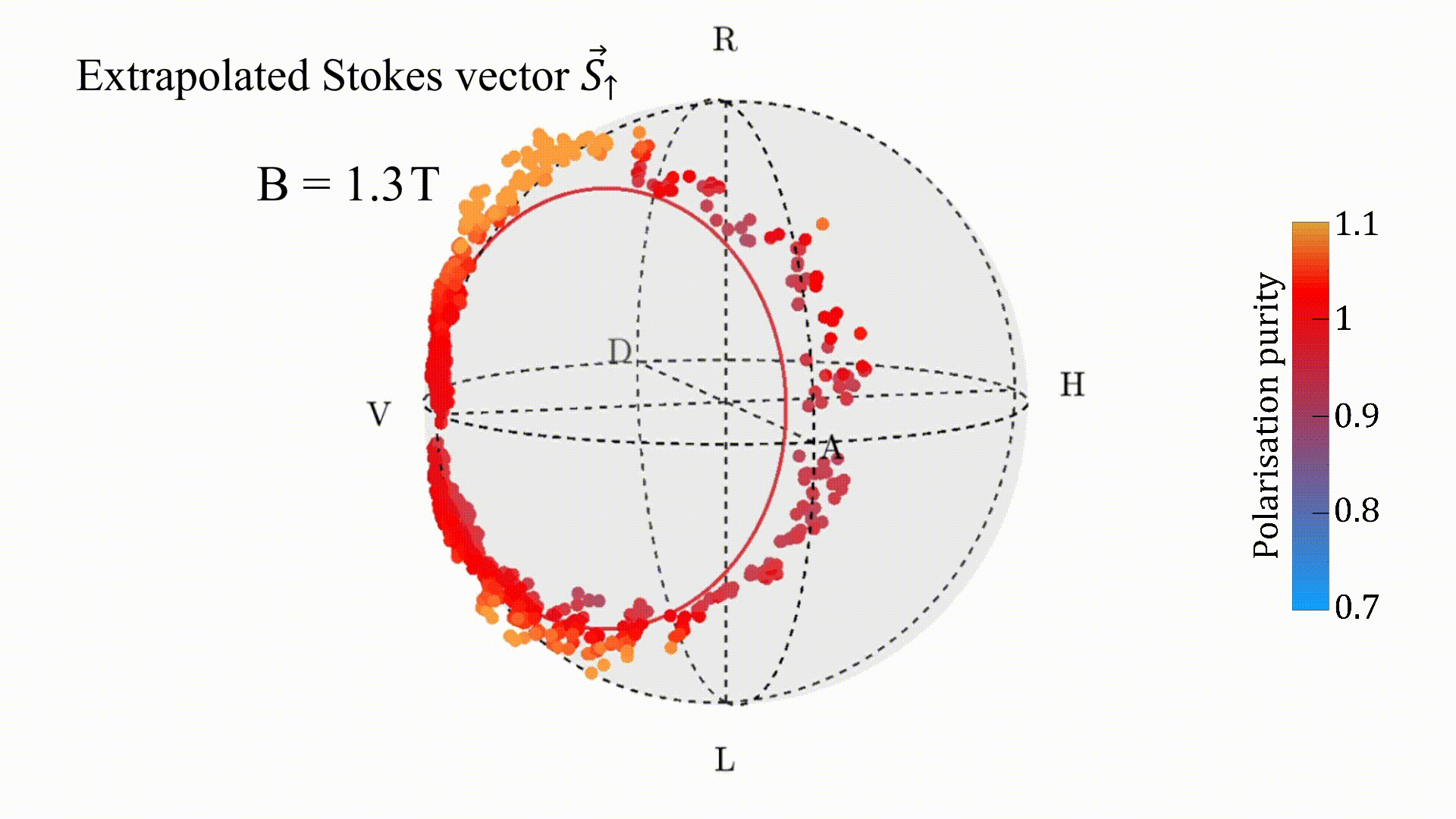

Supplement: Supplementary file 8 — Supplementary Movie 5 [file 41467_2023_44651_MOESM8_ESM.gif]

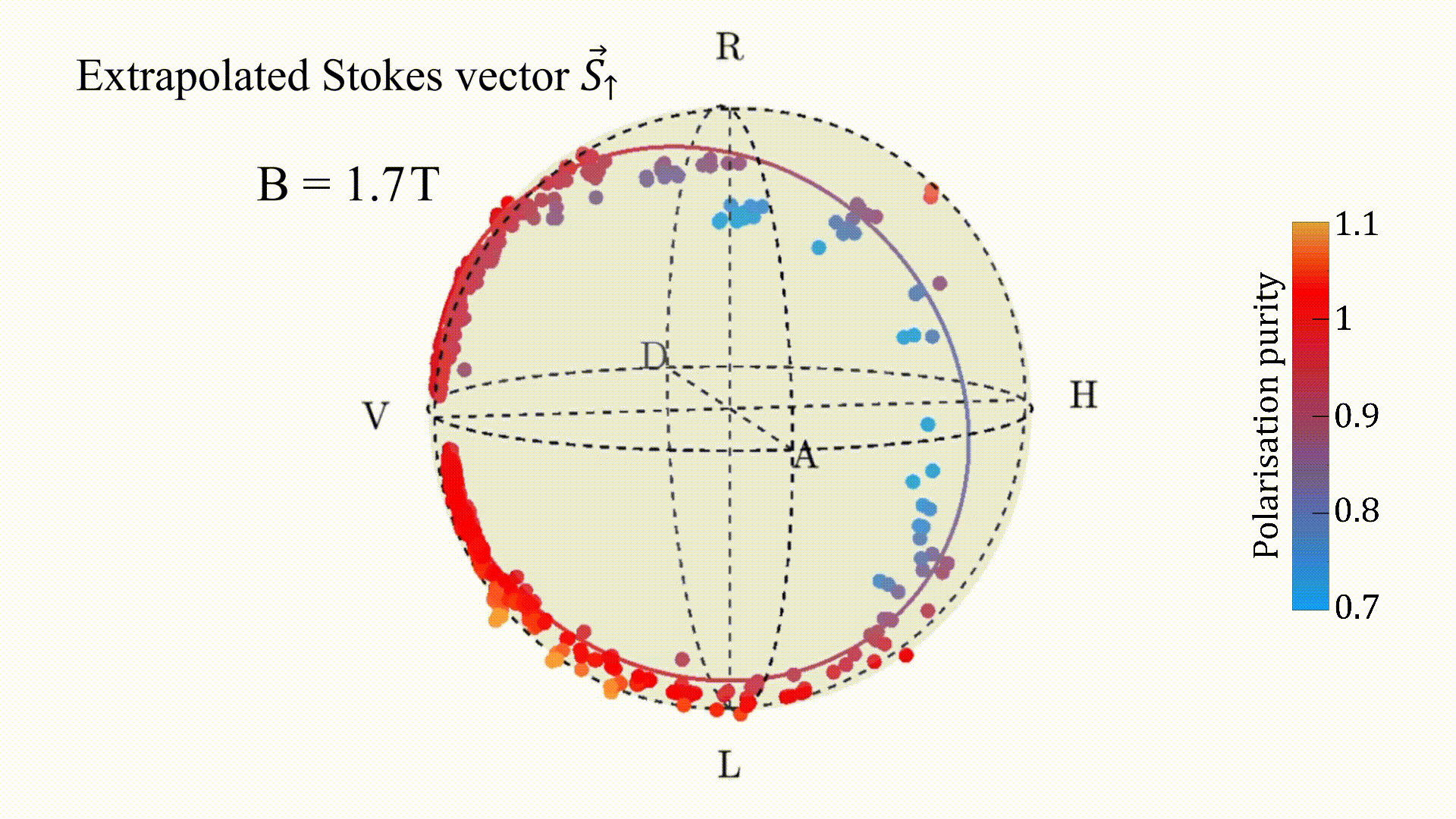

Supplement: Supplementary file 9 — Supplementary Movie 6 [file 41467_2023_44651_MOESM9_ESM.gif]

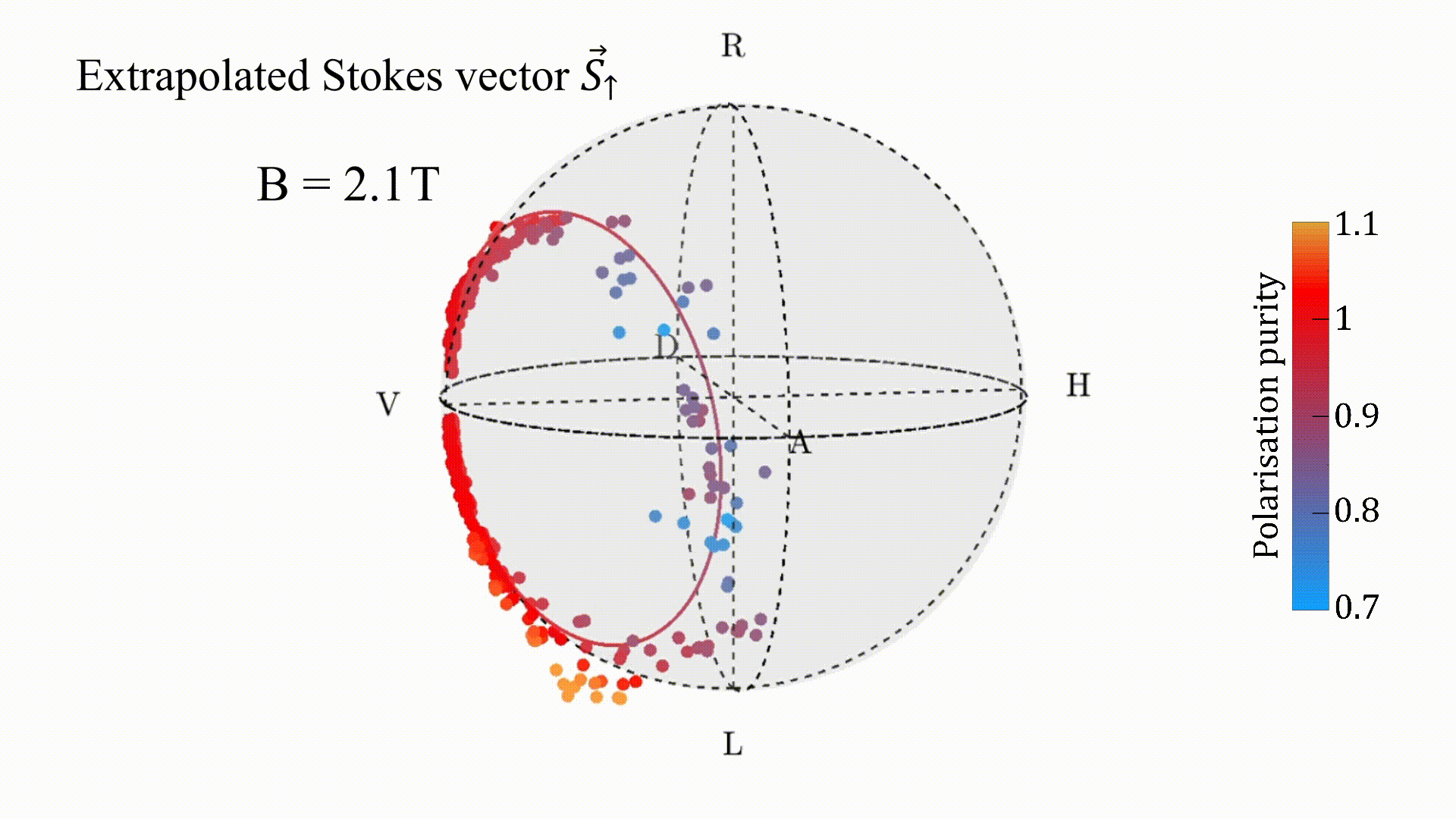

Supplement: Supplementary file 10 — Supplementary Movie 7 [file 41467_2023_44651_MOESM10_ESM.gif]

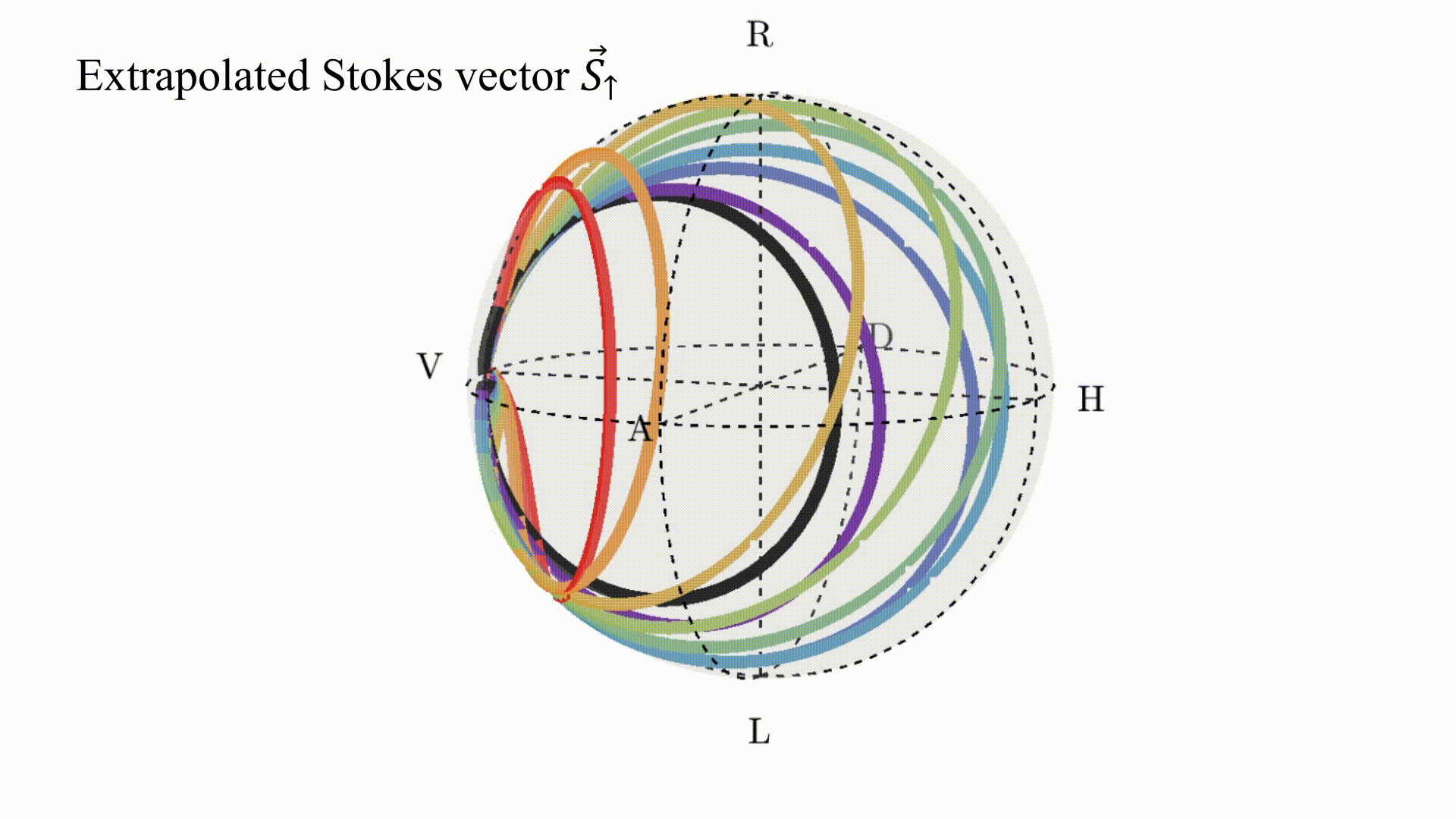

Supplement: Supplementary file 11 — Supplementary Movie 8 [file 41467_2023_44651_MOESM11_ESM.gif]

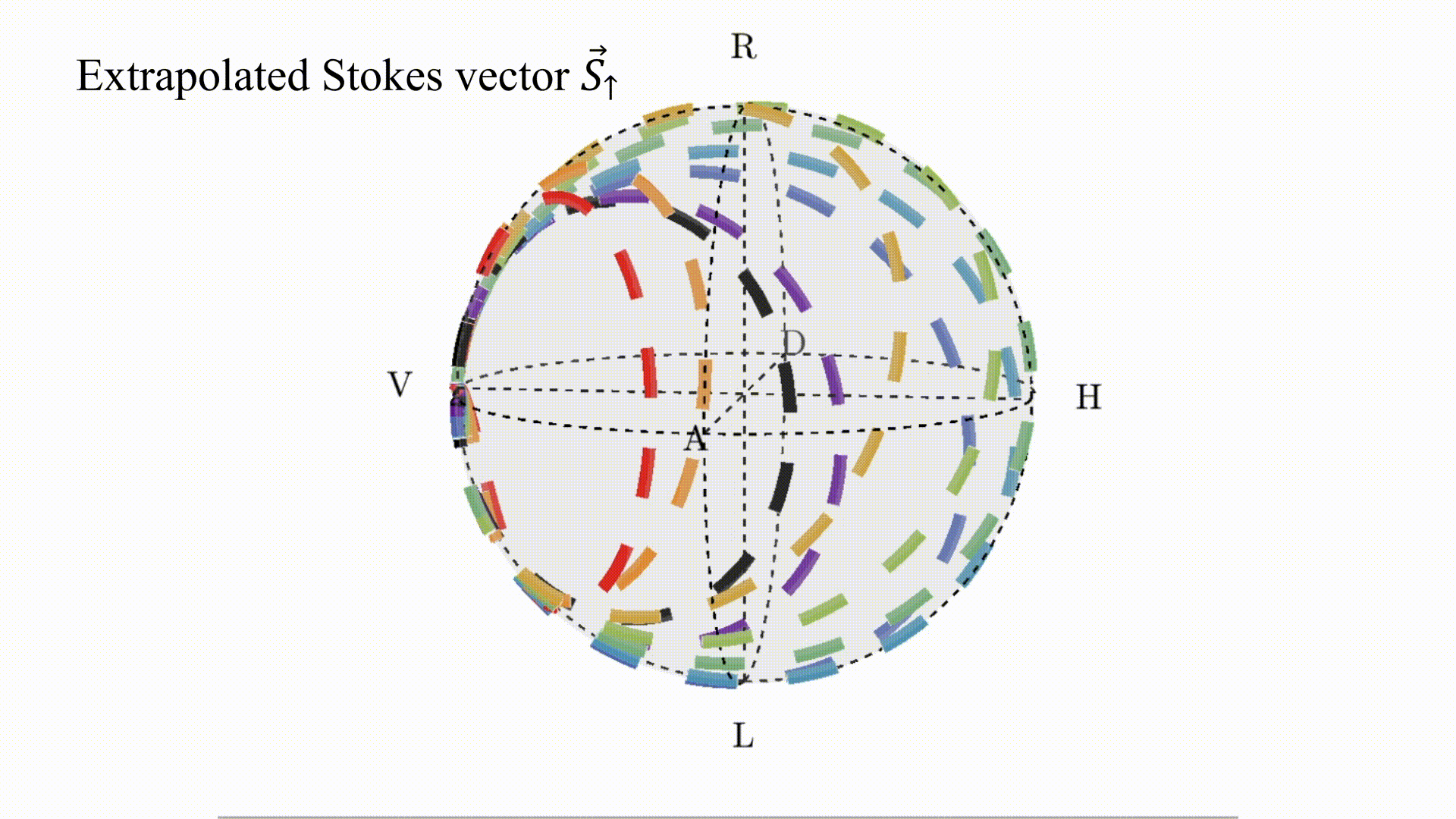

Supplement: Supplementary file 12 — Supplementary Movie 9 [file 41467_2023_44651_MOESM12_ESM.gif]
